# Immunotherapy drug target identification using machine learning and patient-derived tumour explant validation

**DOI:** 10.1038/s42256-026-01201-3

**Published:** 2026-05-18

**Authors:** Marcellus Augustine, Nuno Rocha Nene, Hongchang Fu, Christopher L. Pinder, Lorena Ligammari, Alexander P. Simpson, Irene Sanz-Fernández, Krupa Thakkar, Danwen Qian, Evelyn Fitzsimons, Benjamin S. Simpson, Roberto Vendramin, Andrea Castro, Heather Niederer, Samra Turajlic, Sergio A. Quezada, Nicholas McGranahan, Chris Watkins, Charles Swanton, Kevin Litchfield

**Affiliations:** 1 Tumour Immunogenomics and Immunosurveillance (TIGI) Laboratory, University College London Cancer Institute, London, UK; 2 Cancer Evolution and Genome Instability Laboratory, The Francis Crick Institute, London, UK; 3 Cancer Genome Evolution Research Group, University College London Cancer Institute, London, UK; 4 Cancer Research UK Lung Cancer Centre of Excellence, University College London Cancer Institute, London, UK; 5 Division of Medicine, University College London, London, UK; 6 Department of Statistical Science, University College London, London, UK; 7 Cancer Dynamics Laboratory, The Francis Crick Institute, London, UK; 8 Skin and Renal Unit, Royal Marsden NHS Foundation Trust, London, UK; 9 Cancer Immunology Unit, Research Department of Haematology, University College London Cancer Institute, London, UK; 10 Cancer Research Horizons, The Francis Crick Institute, London, UK; 11 Cancer Dynamics Laboratory, Cancer Research UK Manchester Institute, The University of Manchester, Manchester, UK; 12 Cancer Research UK City of London Centre, University College London, London, UK; 13 Department of Computer Science, Royal Holloway, University of London, London, UK; 14 Department of Oncology, University College London Hospitals, London, UK

**Keywords:** Machine learning, Cancer microenvironment, Tumour immunology

## Abstract

Immunotherapy has revolutionized cancer treatment, yet only a minority of individuals respond clinically, necessitating alternative strategies that can benefit these patients. Novel immuno-oncology targets may achieve this through bypassing resistance mechanisms to standard therapies. We introduce Mining Immunotherapy Drug tArgetS (MIDAS), a multimodal graph neural network system for immuno-oncology target discovery. MIDAS leverages gene interactions, multi-omic patient profiles, immune cell biology, antigen processing, disease associations and phenotypic consequences of genetic perturbations. It generalizes to time-sliced data, outcompetes state-of-the-art baselines (including OpenTargets) and ranks approved targets above those in clinical development. Moreover, MIDAS recovers immunotherapy-response-associated genes in unseen patients, thereby capturing immunotherapy response determinants. Interpretability analyses reveal a reliance on autoimmunity, regulatory networks and immuno-oncology pathways. Functionally perturbing oncostatin M–oncostatin M receptor signalling, a proposed MIDAS target, in TRACERx melanoma-patient-derived explants yielded reduced dysfunctional CD8^+^ T cells, which associate with immunotherapy response, and reduced CCL4 levels. Furthermore, oncostatin M and oncostatin M receptor expression is associated with altered T cell and macrophage profiles in bulk transcriptomic data from patient samples. These data are consistent with a role for oncostatin M–oncostatin M in modulating the tumour microenvironment towards immunosuppressive, tumour-promoting phenotypes. Our results present a machine learning framework for analysing multimodal data for immuno-oncology target discovery.

## Abstract (web summary)

Augustine et al. present a multimodal graph neural network that identifies cancer immunotherapy targets. It distinguishes approved and prospective targets, and promising candidates are validated using a clinically relevant patient-derived platform.

## Main

Checkpoint inhibitor (CPI) immunotherapy has revolutionized cancer treatment, with more than 50 FDA approvals and durable response observed in some patients^1^. However, across cancer types, a minority of patients benefit clinically due to primary and secondary resistance^2,3^. This highlights the urgent need for novel therapies to rescue patients failed by conventional immuno-oncology treatments. CPI response depends on host- and tumour-specific factors^3^, which traditional preclinical models struggle to fully recapitulate, limiting target discovery efforts^4^. The expanding wealth of high-dimensional complex data capturing different tumour–immune facets prompts interest in leveraging machine learning (ML) to derive biological insights^5^. Indeed, sophisticated ML technology capable of modelling disease complexity and inferring mechanistic insights are being increasingly applied to drug discovery across indications^6–9^.

ML target discovery requires training data that richly profile disease mechanisms through different, complementary lenses to recapitulate the biological complexity and variation observed in human patients^10^. Sifting through ever-increasing datasets for highly informative subsets to understand disease mechanisms is a challenging feature selection problem^11^. Integrating domain expertise in system development can address this and enrich for task-specific signals within the training corpus^11^.

Multimodality permits comprehensive disease profiling by capturing complex interactions across omics levels that underlie phenotypes, rather than the mostly correlative insights from isolated omics analyses^8,12–14^. This is particularly relevant for immuno-oncology, where target discovery systems must model host- and tumour-specific factors that influence anti-tumour immune responses. These are studied using diverse assays and data modalities. Multimodal integration facilitates incorporating tumour-intrinsic and tumour-extrinsic modulators of anti-tumour immunity. The former includes antigen presentation^15^ or immune-sensitizing pathway perturbations^3^, whereas the latter includes the tumour microenvironment (TME) composition^3,16,17^. Additional data modalities include causal perturbations driving immune-mediated tumour elimination^18,19^, population-scale gene–phenotype associations^20^ and gene function within broader biological networks^21^. Integrating such multi-omic and functional evidence addresses the limitations of learning discovery engines from largely correlative relationships by providing causal information.

A natural approach to combine multimodal data for gene-centric inference that constrains possible solutions and is supported by previous successes involves encapsulating data within a multimodal biological graph^6–9,22^. Graph neural networks (GNNs) are powerful ML techniques that can be deployed on such graph-structured data to compute node embeddings using message passing frameworks that aggregate input features along routes constrained by network topology.

Here we present a multimodal GNN system, Mining Immunotherapy Drug tArgetS (MIDAS), dedicated to immuno-oncology target discovery and supported by validation in the advanced, clinically relevant patient-derived explant (PDE) experimental platform. We evaluate diverse ML systems on a bespoke, integrated multimodal dataset of patient molecular profiles, preclinical evidence and population-scale genetic data. MIDAS outperforms existing computational and experimental target discovery methods, generalizes to time-sliced data to identify prospective targets, distinguishes approved versus targets in development, and recovers CPI response differentially expressed genes (DEGs). We perturb oncostatin M (OSM)–oncostatin M receptor (OSMR) signalling (a highly ranked MIDAS target) in the ex vivo PDE platform, demonstrating reduced dysfunctional T cells and CCL4 levels, supporting the validity of our approach.

## Results

### Multimodal graph ML system for novel immunotherapy drug target discovery

**Fig. 1:**
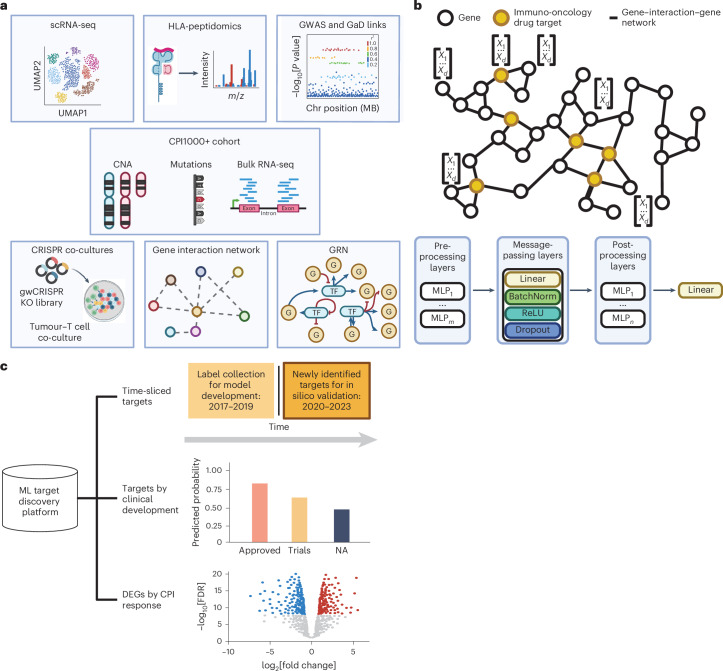
Development of a multimodal graph ML system to identify novel immunotherapy drug targets. **a**, Datasets for immuno-oncology target discovery. GaD, gene–association-disease links from Hetionet. **b**, Graph ML to integrate multimodal biomedical data. GNN message passing aggregates features for each node with its neighbours, as defined by the edge connectivity. For visual clarity, only a subset of nodes have annotated feature vectors, whereas in reality, all nodes will have a corresponding feature vector. *d*, node feature vector length; *m*, number of multilayer perceptron (MLP) pre-processing layers; *n*, number of MLP post-processing layers (Methods). **c**, In silico validation schemes to assess model performance. Schematics created in BioRender; **a**, Augustine, M. http://biorender.com/a3w9xny (2026); **b**, Augustine, M. http://biorender.com/nwtmfo7 (2026); **c**, Augustine, M. http://biorender.com/rhtuz4j (2026).

Therapies acting on targets supported by human genetic evidence are more likely to receive approval^23–25^. To model anti-tumour immunity and harness genetic evidence alongside other omics and non-omics data, we developed MIDAS using a bespoke multimodal immuno-oncology dataset (Fig. 1a,b). This comprised (1) CPI-treated patient exomes and transcriptomes^3^; (2) tumour-infiltrating immune single-cell transcriptomics (single-cell RNA-sequencing (scRNA-seq)) atlases^26,27^; (3) human leucocyte antigen-peptidomics (HLA-peptidomics)^28^; (4) immuno-oncology-relevant gene–phenotype associations^20,21^; and (5) causal gene perturbations from CRISPR tumour–T cell co-cultures^18,19^. Each dataset was pre-processed into low-dimensional representations (Methods and Supplementary Table 1), permitting integration by augmenting the Hetionet gene–interaction–gene (GiG) network^21^.

We developed a binary classification immuno-oncology target discovery framework, applying the ‘closed-world’ assumption in which unlabelled instances are considered negative^7,29^. Positive instances (known targets) represented *n* = 260 non-antigenic targets in clinical development by 2019 (ref. ^30^) (Methods), with remaining genes considered negative. Node classification GNNs were trained to predict the immuno-oncology target status (Fig. 1b).

We defined three in silico validation tasks for the trained model (Fig. 1c). The first assessed generalization to time-sliced data, checking whether the model assigned higher scores for targets entering trials after the target label collection cut-off (in 2019 (ref. ^30^); Methods). Second, owing to high drug development costs^31^, we investigated the prediction translational potential, assessing if the approved targets scored higher than those under clinical development (phases I–III) and remaining genes. Crucially, the model was blinded to these annotations. Third, we assessed CPI response DEG recovery in unseen patients (Methods), probing whether the model captures factors influencing tumour–immune dynamics.

### Graph ML system achieves robust performance across in silico immuno-oncology benchmarks

We investigated multiple GNN architectures for immunotherapy target discovery, varying the *k*-nearest neighbours sampled during message passing to enhance generalization^32^ and efficiency (Methods). All GNNs achieved an area under the receiver operating characteristic (ROC-AUC) > 0.8 on cross-validation (CV) and held-out test folds (Supplementary Fig. 1a and Supplementary Table 2). The highest-performing variants (ranked by the held-out ROC-AUC) were compared using the Bayesian information criterion. A simplified graph isomorphism network (GIN^33^) proved optimal, featuring pre-/post-processing layers that utilise multilayer perceptrons (MLPs), but without MLP-based GIN layers or global concatenation (Supplementary Fig. 1b and Methods). All the following analyses used this model (referred to as MIDAS GIN; Supplementary Table 3).

**Fig. 2:**
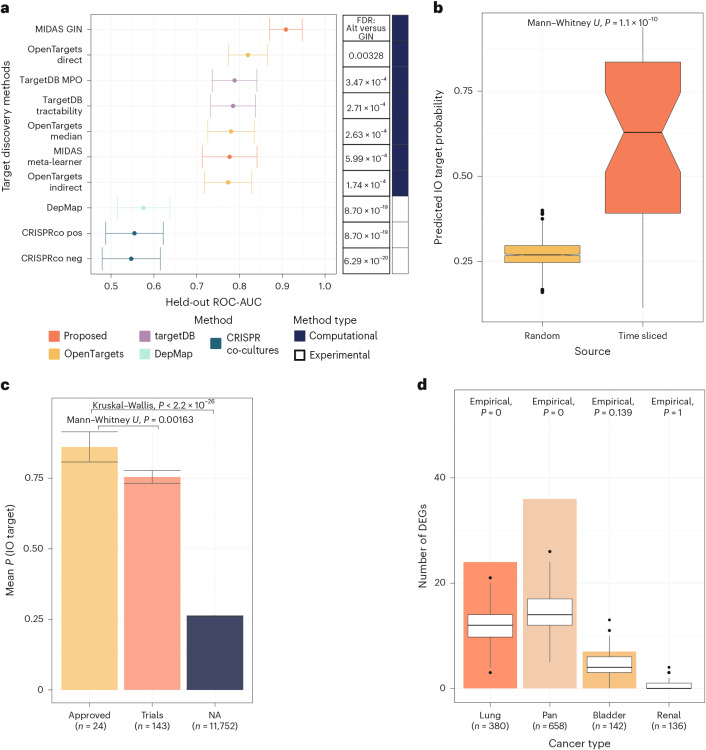
Graph ML system achieves robust performance across in silico immuno-oncology benchmarks. **a**, The proposed graph ML system demonstrates improved performance compared to the tested computational and laboratory target discovery methods. All comparisons were performed on the held-out set of genes to avoid overestimating the performance of our method. The GIN outperformed these existing computational and experimental methods (FDR < 0.05, Delong’s test and bootstrap test for CRISPR co-culture negative MAGeCK scores). Predictions were computed by bagging across CV folds (Methods). ROC-AUC scores are computed using the intersection between genes in the MIDAS held-out set (Methods) and those represented in the alternative methods. CRISPR co-cultures: *n* = 3,819; DepMap: *n* = 3,764; MIDAS GIN: *n* = 2,986; MIDAS meta-learner: *n* = 3,819; OpenTargets direct: *n* = 3,181; OpenTargets indirect: *n* = 3,606; OpenTargets median: *n* = 3,606; TargetDB MPO: *n* = 3,671; TargetDB tractability: *n* = 3,671. Data are presented as the ROC $\pm$ 95% confidence interval. **b**, Framework differentially ranks genes that go on to be addressed in clinical trials in time-sliced data. Predictions were computed by bagging across CV folds (Methods). The time-sliced category contain *n* = 36 targets; the random category represents the mean score for 1,000 randomly sampled sets, each comprising *n* = 36 genes. The *P* value was derived from a two-sided Mann–Whitney *U* test. The centre line indicates the median, box borders represent the upper and lower quartiles, whiskers represent the interquartile range multiplied by 1.5, and black points are outliers. IO, immuno-oncology. **c**, On average, the MIDAS model predicts higher scores for genes that are approved for targeting in clinical practice. NA indicates genes that did not have phase information available. Predictions were computed by bagging across CV folds (Methods). Data are presented as mean values $\pm$ standard error. Exact *P* value for the Kruskal–Wallis test: 4.83 × 10^−70^. The comparison between approved targets and those undergoing clinical trials was assessed using a two-sided Mann–Whitney *U* test. IO, immuno-oncology. **d**, MIDAS identifies genes that are differentially expressed by response status in unseen clinical trials. Comparison of the number of DEGs recovered in the top 200 model predictions (coloured bars) against those found in random (stratified by expression), size-matched gene sets (white box plots). Predictions were computed by bagging across CV folds (Methods). Empirical *P* values were used to assess statistical significance and are shown. The centre line indicates the median, box borders represent the upper and lower quartiles, whiskers represent the interquartile range multiplied by 1.5, and black points are outliers. Source data

To assess whether GNN complexity was necessary, we trained diverse non-geometric models using the same data (swapping GiG topology for node degree; Supplementary Methods; Supplementary Tables 4 and 5 list the performances and optimal hyperparameters, respectively). MIDAS GIN outperformed all variants (false-discovery rate (FDR) < 0.05; Supplementary Fig. 1c). We also implemented a meta-learning approach that sequentially integrated data (Supplementary Methods and Supplementary Fig. 2), combining gene importances from base immunotherapy response predictors (trained using sequencing data) with the remaining features via a stacked binary classifier ensemble. Again, MIDAS GIN proved superior (FDR = 5.99 × 10^−4^; Fig. 2a). Hence, leveraging GNN message passing to integrate multi-omics, preclinical, population-scale genetic data and biological contexts enables improved performance.

We then compared MIDAS GIN against existing target discovery methods (Fig. 2a), finding that it outperformed OpenTargets (https://www.opentargets.org/) direct (FDR = 3.28×10^−3^), indirect (FDR = 1.74×10^−4^) and combined evidence (FDR = 2.63×10^−4^). MIDAS GIN also showed improved performance compared to TargetDB^34^, a random forest (RF) tractability predictor, tractability estimates (FDR = 2.71×10^−4^) and multiparameter optimization (MPO; equally weighing all evidence sources; FDR = 3.47×10^−4^). Additionally, MIDAS GIN performed better than DepMap (https://depmap.org/portal/) gene effect scores (FDR = 8.70×10^−19^), which reflect gene perturbation influences on cell viability, and the CRISPR CD8^+^ T cell co-cultures (FDR = 8.70×10^−19^ and FDR = 6.29×10^−20^).

Next, we assessed the model using our in silico validation tasks. First, MIDAS GIN generalized to the time-sliced data by differentially ranking genes that were prospectively recognized as immuno-oncology targets (*P* = 1.1×10^−10^, Mann–Whitney *U* test; Fig. 2b). OpenTargets direct evidence, the next best performer, also ranked time-sliced targets above random (*P* = 8.7×10^−9^, Mann–Whitney *U* test), but differentiated between them less effectively (Supplementary Fig. 1d).

Second, despite being blinded to clinical phase annotations, MIDAS GIN, on average, predicts higher scores for approved targets compared to non-target genes (Fig. 2c). This result held when comparing approved versus targets in development (*P* = 0.00163, Mann–Whitney *U* test). Recategorizing time-sliced targets as in-development targets did not diminish this (*P* = 2.23×10^−4^, Mann–Whitney *U* test; Supplementary Fig. 1e).

Third, MIDAS GIN recovers CPI response DEGs in unseen trials, even after accounting for gene expression (randomly sampling genes, stratified by mean expression, for the null distribution). The top 200 predictions were enriched for more DEGs than size-matched random sets (empirical *P* < 0.05 in pan- and lung cancers; Fig. 2d). MIDAS GIN correlated with the DEG Wald statistic, with stronger correlations amongst higher-ranked genes observed in pan-, lung and renal cancer (*P* < 0.05, Spearman’s rank; Supplementary Fig. 1f). Hence, MIDAS GIN achieves robust performance, assigns higher scores to prospectively identified immuno-oncology targets as well as to approved targets compared to those in development, and captures patient tumour–immune dynamics, supporting the biological relevance of its predictions.

### Global interpretability analysis reveals features informing immuno-oncology target prediction

**Fig. 3:**
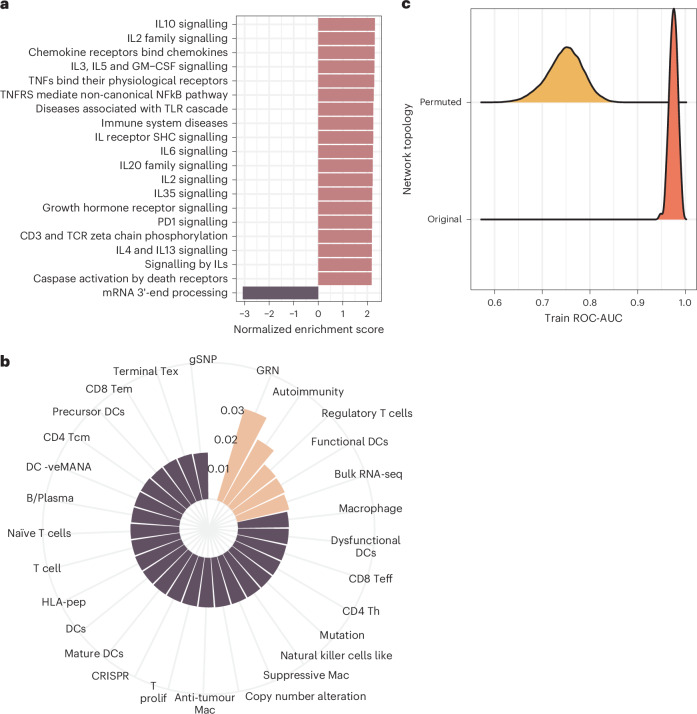
Global interpretability analysis reveals features informing immuno-oncology target prediction. **a**, Modelling framework enriches for immuno-oncology-related pathways. The top 20 pathways enriched amongst the model predictions identified by GSEA. **b**, Transcriptional dynamics and autoimmunity are amongst the data types most influential for the modelling framework. Permutation node feature importance, grouped by biological category, showing how different features contribute to model training. Statistical significance is assessed through empirical *P* values, describing the difference between performance on the training and permuted sets. All features were significantly important (FDR < 0.05). The top five most important features are highlighted. Autoimmunity is used as an umbrella term to include associations with autoimmune, rheumatic and allergic diseases. -veMANA, negative correlation with the mutation-associated neoantigen score; Teff, effector T cells; Th, helper T cells; mac, macrophage; HLA-pep, HLA-peptidomics; Tcm, central memory T cells; Tem, effector memory T cells; Tex, exhausted T cells. **c**, The gene interaction network is critical to model performance. Distribution showing the performance of the trained model when applied to the permuted network compared with performance across all CV folds for predicting known immuno-oncology targets in the training set. Original, the distribution of ROC-AUC in the training set across all 100 CV fold models (*n* = 100); permuted, the distribution of ROC-AUC following 500°-preserving edge permutations for each CV fold model (*n* = 50,000). Source data

We next investigated potential biological drivers of model predictions. Gene Set Enrichment Analysis (GSEA) revealed strong enrichment for immuno-oncology pathways (Fig. 3a, Supplementary Table 6 and Supplementary Note 1), including IL-2, CD3 and TCR signalling. In particular, PD-1 signalling (a canonical immuno-oncology target) was highly enriched, further supporting that MIDAS GIN captures signals relevant to immuno-oncology discovery.

Interpretable drug discovery can help to justify clinical development programme costs^10^ and build a mechanistic understanding. We applied permutation feature importance to assess which factors most influence model training (Methods and Fig. 3b), finding that the gene regulatory network (GRN) and autoimmunity features ranked the highest. Importance rankings were consistent across CV test and held-out sets (Supplementary Fig. 3a,b); the top 4 biological feature categories (14.3%) were stable across splits (Supplementary Fig. 3b). In particular, bulk transcriptomics (RNA-seq) and macrophage biology ranked highly in the CV train but not in the CV test and held-out sets. As a further sensitivity analysis, we examined how feature importance varies with specificity thresholds (measuring the absolute change in recall post-permutation). Again, the top ranked categories remained stable (Supplementary Table 7), highlighting consistent model behaviour. Differences between training and test sets probably reflect features driving minor overfitting, despite robust CV protocols. Since genes were ranked by output scores, and not assigned binary labels, variations across specificity thresholds likely have minimal impact.

Having assessed the influence of node features, we next examined the role of knowledge graph connectivity by permuting GiG edges whilst preserving node degrees, thereby corrupting edge biological information but maintaining node topology. By corrupting GNN message passing mechanics, network permutation caused the greatest training performance drop (Fig. 3c), underscoring the critical role of gene–gene interactions for immuno-oncology discovery.

### MIDAS identifies candidate immunotherapy drug targets

To identify novel immuno-oncology targets, we removed positively labelled instances from the top 300 predictions, and reviewed the remaining for novelty and biological plausibility, shortlisting 43 targets (Methods and Supplementary Table 8). These were manually reviewed, in ranked order, for biological plausibility, safety, druggability, clinical and preclinical competition, and availability of appropriate compounds for ex vivo validation.

**Fig. 4:**
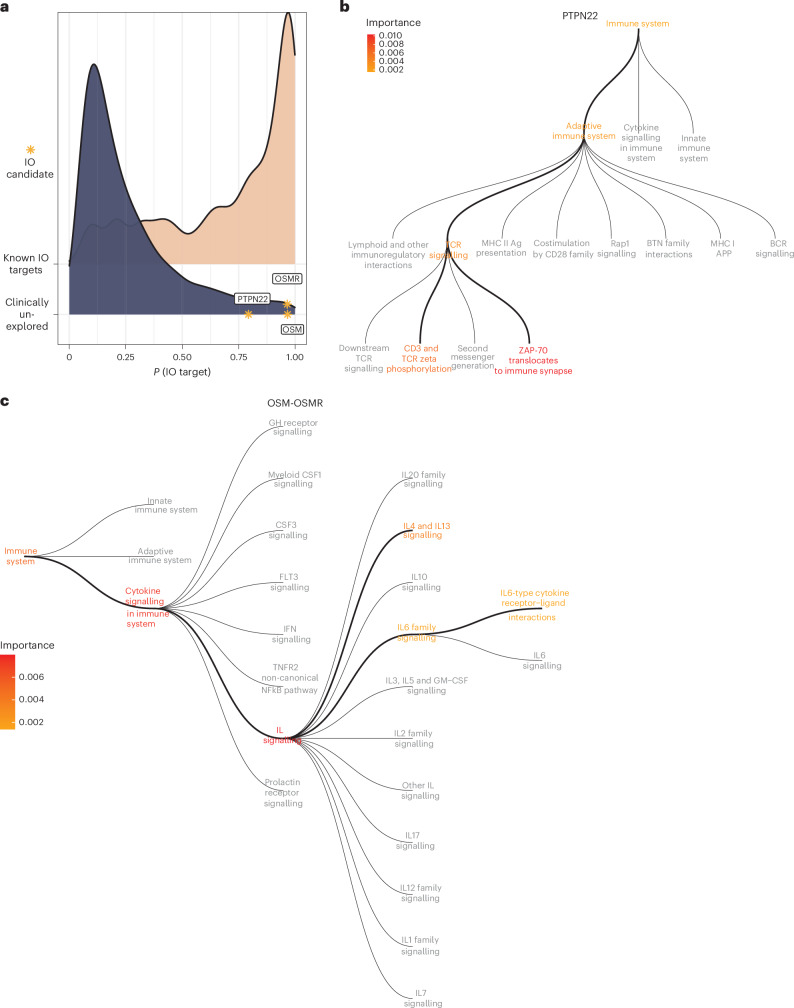
Novel candidate immunotherapy drug targets. **a**, OSM and OSMR are novel candidate immunotherapy drug targets. For comparison purposes, the predicted probability for PTPN22 (a previously suggested preclinical target), and a density plot showing the predictions for known immuno-oncology targets and non-targets are provided. **b**, Pathway-based permutation importance for PTPN22. Colour bar indicates the importance of manually curated pathways for predicting PTPN22 as a drug target. APP, antigen processing and presentation. **c**, Pathway-based permutation importance for the OSM–OSMR axis. Colour bar indicates the importance of manually curated pathways for predicting the OSM–OSMR axis as an immunotherapy candidate. In **b** and **c**, the grey pathways are from the Reactome pathway hierarchy and were not perturbed either because the target does not belong to that pathway or because there were insufficient edges to permute. Source data

Proposed immunotherapy targets include OSM and OSMR, which score 0.966 and 0.965, respectively (Fig. 4a). OSM, an IL-6 cytokine family member produced by activated T cells and macrophages, acts on fibroblasts, cancer and myeloid cells. OSMR dimerizes with IL-31RA or IL-6ST (gp130) to mediate signalling^35^. Another highly predicted candidate, *PTPN22* (scoring 0.793), is a tyrosine phosphatase that negatively regulates TCR signalling (Fig. 4a). Preclinical evidence (disjoint from the MIDAS training data) supports both candidates, underscoring the effectiveness of MIDAS GIN^36–42^.

Next, we investigated the factors driving candidate prediction using a permutation approach (Supplementary Fig. 4a,b and Methods). Targets were mapped to pathways, which were independently permuted, defining importance as the normalized absolute prediction change. Key pathways for *PTPN22* are functionally relevant and include those used in TCR signalling, specifically ZAP-70 translocation as well as CD3 and TCRζ phosphorylation (Fig. 4b). Downstream of the TCR, PTPN22 dephosphorylates TCRζ, CD3ε and ZAP-70 (ref. ^43^). ZAP-70 phosphorylation correlates strongly with CD8^+^ T cell activation markers, including granzyme B (GZMB) and Ki-67 (ref. ^39^). Consistently, inhibiting PTPN22 in mice promotes CD8^+^ T cell-mediated anti-tumour responses^37,39^. Hence, MIDAS GIN likely scores PTPN22 highly by leveraging relevant functional biology.

Pathway importances were averaged across the interacting partners, OSM and OSMR. Interleukin and cytokine signalling were the most influential (Fig. 4c), showcasing a dependence on immune function. The preferential reliance on IL-4 and IL-13 signalling could reflect that OSM induces IL-4 and IL-13 (ref. ^44^). In turn, IL-4 promotes IL-31RA expression, which heterodimerizes with OSMR^45,46^, and myeloid-mediated immunosuppression^47,48^. Thus, MIDAS likely deems OSM–OSMR signalling as an immunotherapy target based on relevant tumour immunology.

### Functional validation of novel immunotherapy drug targets

**Fig. 5:**
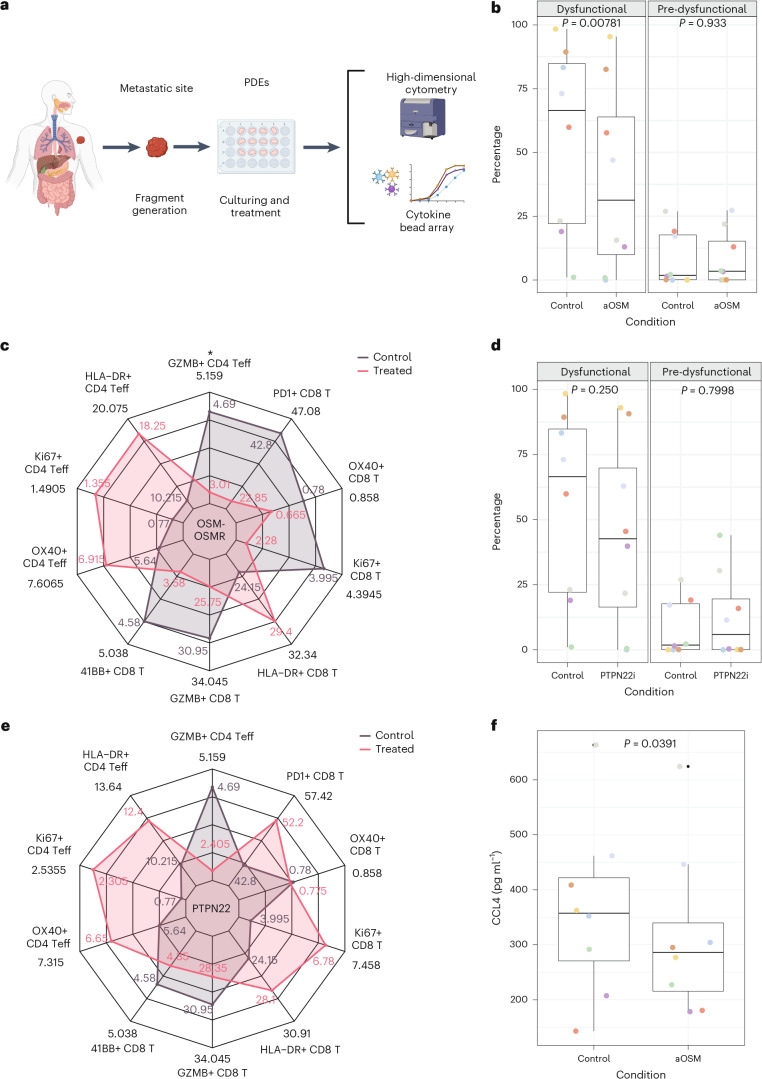
Functional validation of novel drug targets. **a**, Schematic showing the PDE experimental validation platform. All experiments used *n* = 8 melanoma PDEs, with dimethyl sulfoxide (DMSO) being used as the control condition. **b**, Effect of perturbing OSM–OSMR on dysfunctional (PD-1^+^TCF-7^−^CD39^+^) and pre-dysfunctional (PD-1^+^TCF-7^+^CD39^−^) CD8^+^ T cells. Points are coloured by sample. *P* values from a paired two-sided Wilcoxon signed-rank test are shown. Data represent *n* = 8 melanoma PDEs. The centre line indicates the median, box borders represent the upper and lower quartiles, and whiskers represent the interquartile range multiplied by 1.5. **c**, Changes observed in CD4^+^ and CD8^+^ functional markers following OSM–OSMR blockade. * indicates *P* < 0.05. Exact *P* value for the significant change observed in CD4^+^GZMB^+^ Teff: *P* = 0.0156, paired two-sided Wilcoxon signed-rank test. Data represent *n* = 8 melanoma PDEs. Numerical values annotated on the plot represent the median. On the radar plot, for visual clarity, the maximum annotated value = maximum value of that quantity + (maximum value of that quantity × 10%) and the minimum point = minimum value of that quantity – (minimum value of that quantity × 10%). The *y* axis represents the percentage of parental population (Supplementary Fig. 11 shows the gating strategy), with the maximum and minimum points defined as above. **d**, Inhibiting PTPN22 did not significantly alter dysfunctional (PD-1^+^TCF-7^−^CD39^+^) or pre-dysfunctional (PD-1^+^TCF-7^+^CD39^−^) CD8^+^ T cells. Points are coloured by sample. Data represent *n* = 8 melanoma PDEs. *P* values from a paired two-sided Wilcoxon signed-rank test are shown. The centre line indicates the median, box borders represent the upper and lower quartiles, and whiskers represent the interquartile range multiplied by 1.5. **e**, Changes in CD4^+^ and CD8^+^ T cell functional markers following the inhibition of PTPN22. Data were analysed using a paired two-sided Wilcoxon signed-rank test and no significant changes were observed. Data represent *n* = 8 melanoma PDEs. Numerical values annotated on the plot represent the median. On the radar plot, for visual clarity, the maximum annotated value = maximum value of that quantity + (maximum value of that quantity × 10%) and the minimum point = minimum value of that quantity – (minimum value of that quantity × 10%). The *y* axis represents the percentage of parental population (Supplementary Fig. 11 shows the gating strategy), with the maximum and minimum points defined as above. **f**, CCL4 expression level by cytokine bead array in *n* = 8 melanoma PDEs following anti-OSM treatment. *P* value, from a paired two-sided Wilcoxon signed-rank test, for the difference in CCL4 expression between anti-OSM and control = 0.0391. The centre line indicates the median, box borders represent the upper and lower quartiles, whiskers represent the interquartile range multiplied by 1.5, and black points are outliers. Illustration in **a** created in BioRender; Augustine, M. http://biorender.com/k5w99jf (2026). Source data

To evaluate predicted targets, we independently blocked OSM–OSMR and PTPN22 signalling (with an anti-OSM antibody and PTPN22 inhibitor, respectively) in *n* = 8 stage 3 or 4 melanoma PDEs, assessing for an impact on T cell phenotypes and functionality (Fig. 5a and Supplementary Tables 9 and 10). PDEs are a sophisticated preclinical platform that preserves the endogenous TME, facilitating the investigation of functional perturbations in tumour-infiltrating immune cells^4^. They recapitulate patient CPI response^49^ and have been used to probe new immunotherapies^50–55^.

Since PTPN22 acts downstream of the TCR, we expected its inhibition to directly affect T cells. By contrast, anti-OSM was expected to operate indirectly, blocking T cell/macrophage-produced OSM signalling in OSMR-expressing cells (fibroblasts, cancer and myeloid cells), which may subsequently influence T cell states. Using high-dimensional flow cytometry, we profiled immune phenotypes in CD4^+^ and CD8^+^ T cells, assessing markers of proliferation (Ki-67), cytotoxicity (GZMB) and activation (HLA-DR, OX40 (CD134), 4-1BB (CD137) and PD-1 (CD279)). We also investigated pre-dysfunctional (PD-1^+^TCF-7^+^CD39^−^) and dysfunctional (PD-1^+^TCF-7^−^CD39^+^) CD8^+^ T subsets, which correlate with the CPI response^56^.

Following anti-OSM treatment, we observed reduced dysfunctional CD8^+^ T cells (PD-1^+^TCF-7^−^CD39^+^; *P* = 0.00781; Fig. 5b), no effect on pre-dysfunctional CD8^+^ T cells (*P* = 0.933; Fig. 5b) and a decrease in CD4^+^GZMB^+^ T effectors (*P* = 0.0156; Fig. 5c). Upon PTPN22 inhibition, we noted a decrease in dysfunctional CD8^+^ T cells (Fig. 5d) and an increase in CD4^+^ T effectors expressing Ki-67, HLA-DR, or OX40 (CD134) and CD8^+^ T cells expressing Ki-67, HLA-DR, or PD-1 (Fig. 5e), although none of these achieved statistical significance.

Cytokine bead array (CBA) analysis of culture medium from these experiments revealed reduced CCL4 following anti-OSM treatment (*P* = 0.0391; Fig. 5f). Additionally, the *CCL4* expression correlates with *OSM* in lung (Spearman *ρ* = 0.368, *P* = 1.30×10^−13^), bladder (Spearman *ρ* = 0.518, *P* = 3.99×10^−11^) and renal (Spearman *ρ* = 0.271, *P* = 0.00141) cancers, and with *OSMR* in bladder cancer (Spearman *ρ* = 0.494, *P* = 6.48×10^−10^), in the CPI2500 cohort (paper in preparation). Since CCL4 is associated with tumour-promoting macrophages^57^, this is consistent with a potential role for OSM–OSMR signalling in polarizing macrophages towards M2-like phenotypes (associated with immunosuppressive, tumour-promoting effects).

To further explore its immune associations, we next examined whether *OSM* and *OSMR* gene expression associates with CIBERSORT T cell scores^58^, which reflect the estimated relative proportions of T cell subtypes of interest, in CPI2500 tumour bulk transcriptomes (paper in preparation). High *OSM* and *OSMR* were associated with decreased CD4^+^ resting memory, but increased CD4^+^ activated memory, CD8^+^ and regulatory T cells (Supplementary Figs. 5 and 6). Signature resolution precluded CD8^+^ T subtype analysis, meaning it was not possible to confirm whether this reflected an increase in exhausted cells.

Given the link between OSM–OSMR and suppressive M2-like macrophage polarization^40–42,59–64^, we investigated CIBERSORT macrophage quantifications^58^. A trend towards increased M2 macrophages in bladder cancer was observed at a higher expression of *OSMR* (*P* = 0.0281, FDR = 0.112; Supplementary Fig. 7) and *OSM* (*P* = 0.0720, FDR = 0.144; Supplementary Fig. 8). Elevated M1 macrophages (a pro-inflammatory phenotype associated with increased anti-microbial and anti-tumour activity) associated with higher *OSMR* (FDR = 0.0467; Supplementary Fig. 7) and *OSM* (FDR = 0.00357; Supplementary Fig. 8) expression in bladder cancer. An increase in the M1/M2 ratio at higher *OSM* expression was found in lung cancer (FDR = 0.0215; Supplementary Fig. 8). M0 macrophages (naïve macrophages that undergo polarization towards M1- or M2-like phenotypes) were elevated at higher levels of *OSMR* (bladder: FDR = 0.0467; Supplementary Fig. 7) and *OSM* (bladder: FDR = 0.00357; lung: FDR = 9.5×10^−5^; renal: *P* = 0.050, FDR = 0.121; Supplementary Fig. 8). These data suggest that OSM–OSMR signalling may contribute to influencing TME macrophage phenotypes.

Although PTPN22 inhibition did not significantly alter the tumour-infiltrating T lymphocyte compartments, a small decrease in CD4^+^ T cell cytotoxicity and a reduction in dysfunctional CD8^+^ T cells (a population associated with immunotherapy response) were observed following OSM-OSMR perturbation. These findings support OSM–OSMR blockade as a candidate immunotherapy strategy, underscoring the utility of MIDAS for novel immuno-oncology target discovery.

## Discussion

We propose MIDAS, a multimodal GNN system, as a dedicated and effective cancer immunotherapy target discovery engine. It achieves robust performance at recovering immuno-oncology targets, outperforming existing tested computational and experimental methods (including OpenTargets and CRISPR co-cultures). MIDAS generalizes to time-sliced data, differentially scoring targets that entered clinical trials prospectively as well as targets that are approved compared to those in development, which suggests important ramifications for derisking highly expensive, failure-prone drug discovery programmes^31,65^. Our experiments highlight the advantage of using GNNs over alternative ML frameworks, as they effectively leverage the underlying gene interaction network, which contains information central to target discovery. Furthermore, MIDAS GIN enriches for fundamental tumour immunology (Supplementary Note 1) and its predictions can be influenced by relevant biological pathways, showing that it can extract pertinent information from the input multimodal dataset and lending credence to its predictions.

MIDAS GIN scores the *OSM* and *OSMR* genes highly. They were previously implicated in tumour-promoting TMEs in mouse pancreatic and breast cancer models^40–42^. Extending this, we observe significantly reduced dysfunctional CD8^+^ T cells and CCL4 levels (linked to tumour-promoting macrophages^57^) following OSM-OSMR perturbation in a clinically relevant experimental platform that retains the TME, recapitulates patient response and has facilitated immunotherapy exploration across cancers^49–55^.

*OSM* and *OSMR* expressions are associated with altered T cell profiles. In particular, the increased proportions of regulatory T cells are consistent with prior reports, showing less exhausted intratumoural T cells in OSM-deficient tumours^41^. Although both M1 and M2 macrophages were elevated in bladder cancer, the elevated M0 macrophage scores noted with higher expressions of *OSM* and *OSMR* genes could indicate polarization away from anti-tumour M1-like tumour-associated macrophages, consistent with a role for OSM–OSMR in promoting tumour-associated macrophage infiltrates^40–42,59–64^. These data imply that OSM–OSMR may act in a functional capacity within the TME, thereby underscoring the efficacy of MIDAS GIN.

Anti-OSM agents have been trialled in autoimmune and rheumatological indications, but none have achieved FDA approval. Despite some favourable safety data in phase I trials (ClinicalTrials.gov ID NCT04138043 (ref. ^66^)), poor safety profiles and lack of efficacy were observed during phase II (refs. ^67,68^). We propose that future work should focus on oncology indications and aim to address the existing issues regarding binding affinity^68^ and safety. The multiple agents in preclinical development^69^ indicate continued interest in therapeutically manipulating this axis.

GNNs^70,71^ and other graph-based approaches^7^ have advanced the integration of multi-omic datasets in biology, attracting considerable interest, particularly regarding their explainability. However, no algorithm achieves superiority across all evaluation dimensions, invariably necessitating trade-offs^72^. Due to the size and density of the underlying graph used in the MIDAS GIN model, along with its complex, multilayered structure, executing a GNN-specific interpretability routine would require benchmarking several models, which is computationally prohibitive for our main objectives. Moreover, current interpretability methods lack an extensively validated and accepted framework regarding appropriate performance metrics^73^. We, therefore, utilized a permutation strategy to explore how node features and GiG network topology influence MIDAS GIN.

Transcriptional dynamics (GRN) were the most important, possibly echoing that immuno-oncology targets modulate immune cell transcriptional states^74^ towards less exhausted, more anti-inflammatory phenotypes. The reliance on autoimmunity coincides with the link between it and immuno-oncology^75–77^, as gene perturbations driving immune hyperactivity to cause disease could feasibly trigger anti-tumour immunity.

Another key feature alludes to the role genes play within Treg and functional dendritic cell (DC) interactomes. Regulatory T cells suppress cytotoxic CD8^+^ T cells and restrain anti-tumour immunity, rendering them an immunotherapy target^78,79^. Functional DCs present tumour-derived antigen to naïve T and B cells, connecting innate and adaptive immunities. They cross-link CD4^+^ and CD8^+^ T cells, which is critical for maximal CD8^+^ cytotoxicity, with these triads linked to the CPI response^80^. Together, these data imply that MIDAS GIN is probably driven by relevant tumour immunology.

In future, MIDAS GIN embeddings or output scores could be augmented with new information (for example, additional scRNA-seq or CRISPR screen data) to enrich for targets with specific traits. This could facilitate flexible use of model insights without retraining the full model, reducing resource demands.

Despite these successes, MIDAS has limitations. First, posing target discovery as a binary classification problem assumes all genes addressed in clinical trials are true, and equal, immunotherapy targets. This expands the positive set for training, but identifying targets that will probably receive regulatory approval is more efficient due to clinical trial attrition and development costs^31,65^.

Second, unlabelled genes were treated as negatives, a standard assumption in target discovery methods^7^, as proving a gene has no relevance to the domain of interest is not trivial. This introduces noise as undiscovered immunotherapy targets are mislabelled. Yet, using undersampling of negatively labelled nodes, repeated CV and fold-model bagging ensured that MIDAS aligned with robust ensemble-based positive-unlabelled learning methods^29,81,82^. Additionally, MIDAS GIN leverages biological context and network connectivity, which acts as prior knowledge for each class, to identify candidate immunotherapy targets, following methodologies proposed for advanced negative sampling in positive-unlabelled learning frameworks for knowledge graphs^83^.

Third, manual review was required to triage targets and decide whether they should be perturbed via agonistic or antagonistic strategies, which can introduce bias. Future work should focus on these areas to create true end-to-end ML target discovery pipelines. Since current approaches output predictions for thousands of genes, a systematic functional assessment of high-scoring candidates would incur prohibitively large time and financial costs. This could be addressed through, for example, encoding druggability predictions, protein structure information or literature-derived features within the input features. Regarding perturbation direction, strategies could include incorporating target directionality in the classification task, or explainability approaches that infer whether a candidate is considered stimulatory or inhibitory.

The success of MIDAS across in silico and functional validation tasks staunchly supports applying sophisticated ML for immuno-oncology target discovery. It underscores the value of multimodal, multi-omics datasets to accurately model factors influencing tumour–immune dynamics. Leveraging tools such as MIDAS to produce data-driven candidates could ameliorate the financial burden, long timescales and high attrition that plague clinical trials and traditional target discovery.

## Methods

### Datasets

#### CPI1000+ cohort

The CPI1000+ cohort comprised 15 studies and 9 tumour types (melanoma, lung, bladder, renal, breast, gastric, colorectal cancer, head and neck, with the remaining tumour types grouped under the “other” category). Whole exome and bulk transcriptomics (RNA-seq) sequencing data were processed as previously described^3^. Clinical end-points were defined by radiological response according to the RECIST criteria. Complete response (CR) or partial response (PR) was classified as a responder (R), whereas stable disease (SD) or progressive disease (PD) was classed as a non-responder (NR). Sample numbers were *n* = 941 (R = 259, NR = 682; mutation), *n* = 1,061 (R = 276, NR = 785; copy number alteration) and *n* = 933 (R = 249, NR = 684; RNA-seq) patients.

Bulk RNA-seq transcripts per million data were quantile normalized using the preprocessCore R package (v.1.56.0)^84^, after excluding genes unexpressed in at least 65% of patients. The effects of the source study and tissue source (whether fresh frozen or fresh frozen and paraffin embedded) were regressed out using linear regression.

The processed exome data were categorized for modelling as follows (Supplementary Table 1): mutation data were classified into loss of function, missense or otherwise (non-mutated/synonymous mutation), with ties broken according to this hierarchy. Copy number data were represented using log[R] (defined as $\log [{\rm{R}}]=\frac{{\log }_{2}[\mathrm{total}\,\mathrm{copy}\,\mathrm{number}]}{2}$).

#### scRNA-seq datasets

We utilized previously constructed and internal scRNA-seq atlases specific to B cells^26^, T cells and DCs, together with a version of a published macrophage atlas^27^. Processed data in the form of raw counts were obtained from the Gene Expression Omnibus under the following accession numbers: GSE123813, GSE121638, GSE131907, GSE123139, GSE114727, GSE127465, GSE178341, GSE148071 and GSE169246. Additionally, raw count data were downloaded from the Sequence Read Archive with the accession number SRZ190804 (ref. ^85^) from https://github.com/czbiohub-sf/scell_lung_adenocarcinoma (ref. ^86^), and from http://blueprint.lambrechtslab.org (ref. ^87^). Further, additional information was sourced from ref. ^88^. Processed scRNA-seq data from two additional cohorts were retrieved from the Single Cell Portal (https://singlecell.broadinstitute.org/single_cell/study/SCP1288/tumor-and-immune-reprogramming-during-immunotherapy-in-advanced-renal-cell-carcinoma#study-summary) and the Human Tumor Atlas Network data portal at https://data.humantumoratlas.org/ (ref. ^89^). An additional scRNA-seq dataset was requested from the corresponding author of ref. ^90^.

For each atlas, cells of the specific lineage were extracted from each scRNA-seq study based on the annotations provided in the source publications. The raw count matrices were merged and analysed using Seurat (v.4.0.6)^91^. Quality control was rigorously applied to the cells based on several quality control metrics, including total unique molecular identifier count, total number of expressed genes and the percentage of mitochondrial gene expression. Cells that did not meet the following criteria were filtered out: (1) 300 < unique molecular identifiers < 5,000,000, (2) 200 < expressed genes < 6,000 and (3) mitochondrial gene expression percentage < 20%.

The remaining cells were normalized using the SCTransform function from Seurat (v.4)^92^. Dimensional reduction via canonical correlation analysis was used to identify anchors using 3,000 genes, which were then used by the IntegrateData function to eliminate batch effects. Principal component analysis was performed on the integration-transformed expression matrix, and the top 30 principal components were used for graph-based clustering and further dimensionality reduction using uniform manifold approximation and projection (UMAP).

Clusters were identified using the FindClusters function^92^, with the resolution parameter varied from 0 to 1. Final resolutions were determined to be 0.5 for macrophages, 0.4 for DCs and T cells and 0.2 for B cells, selected based on the elbow plot method. The FindAllMarkers function from Seurat^92^ was used for intercluster differential expression analyses. The top cluster-specific DEGs were subsequently utilized to assign cell-type labels to the clusters.

#### Cell-type-specific interactomes

SCINET (v. 1.0)^93^ was applied to scRNA-seq data normalized using SCTransform (from the Seurat package^91,92^) to construct cell-type-specific protein–protein interaction networks (Supplementary Table 1). SCINET was not used for batch correction. The resulting interactomes were validated by assessing whether cell-type marker genes, comprising the top 50 DEGs (or all DEGs for cell types with <50 DEGs), had higher topological specificity scores than non-marker genes (Supplementary Fig. 9).

#### EDGE immunopeptidomics cohort

Publicly available matched HLA-peptidomics and bulk transcriptomics data (*n* = 74 samples, where *n* = 60 were from patients diagnosed with cancer) were downloaded^28^ and subsequently processed to yield Pearson correlation coefficients describing associations between gene expression and peptide presentation across all sample HLA molecules (Supplementary Table 1). A *q*-value threshold of 0.05 was used to identify peptides that were detected on the HLA molecules from a given sample.

#### GWAS catalogue

Germline single-nucleotide polymorphism (SNP) data were downloaded from the genome-wide association studies (GWAS) catalogue^20^. Intergenic SNPs were excluded. Significant SNP–phenotype associations (using the Bonferroni-corrected threshold: 5×10^−8^) for immuno-oncology-relevant phenotypes were extracted^77^. Next, they were categorized, using domain expertise, into autoimmune/rheumatic/allergic (including diseases from ref. ^94^), blood counts and cytokine/chemokine levels. For each gene, the number of SNPs associated with each of these categories was counted and used as features for downstream immuno-oncology target discovery (Supplementary Table 1).

#### Genome-wide CRISPR co-culture screens

Genome-wide CRISPR screens derived from publicly available tumour–T cell co-culture screens^18,19^ were downloaded and processed using MAGeCK with default values^95^. These results were represented as –log_10_(MAGeCK scores) and indicate whether gene knockouts in cancer cells led to proliferation (immune evasion) or death (immune sensitization) when co-cultured with T cells (Supplementary Table 1). Features corresponded to immune-evading and -sensitized cells, for each cell line separately.

### Pathway analyses

Over-representation and GSEA analyses were performed using the WebGestaltR package (v.0.4.6)^96^, with the Reactome pathway database^97^ and adjusting for multiplicity using the FDR (Benjamini–Hochberg) method, with significance defined as FDR < 0.05. Over-representation analyses were performed to identify processes that were upregulated in a pre-specified list of interest, whereas GSEA was used when there was no prior filtering of genes.

### Statistical analysis

All statistical tests performed were two sided, unless otherwise stated. Multiple testing correction was performed using the FDR. The threshold used for significance was either *P* = 0.05 or FDR = 0.05, if multiple testing adjustment was performed. Normality was assessed using either the Shapiro–Wilk test or the Anderson–Darling test (if sample size was more than 5,000). Unless stated otherwise, parametric tests were used only if the data were normally distributed based on the above tests, with non-parametric methods used otherwise. Comparisons of ROC-AUC were performed using the DeLong’s test or bootstrapping, in the case of curves with different directions, using the pROC (v.1.18.5) R package^98^.

In certain cases, empirical *P* values were calculated from permutation tests using the definition of a *P* value, the probability of obtaining a value at least as extreme as that observed: empirical *P* $=\frac{{\rm{number\; instances}}\ge {\rm{|observed\; value|}}}{{\rm{total\; number\; of\; instances}}}$. *P* values obtained in this manner are explicitly stated in text.

No a priori power calculations were performed, and blinding was not used. Held-out sets were not used for model training or hyperparameter optimization. Unless otherwise stated, all statistical analyses were performed in R (v.4.1.3).

### CIBERSORT analysis

Bulk RNA-seq data from the CPI2500 cohort, an extension of our previous CPI1000+ cohort^3^, were processed using a standardized in-house bioinformatics pipeline developed within the CPI2500 working group. Gene expression quantification was performed using RSEM^99^, producing transcripts per million values for all annotated genes. For immune cell composition analysis, CIBERSORT^58^ was applied to the expression data to estimate the relative proportions of 22 immune cell types. Only samples with successful deconvolution results (*P* < 0.05) were retained for downstream analysis. All correlation analyses of the CPI2500 data were assessed via the Spearman’s rank correlation coefficient using transcripts per million values within each cancer type. Comparisons of estimated proportions of specific immune cell types between samples with high and low *OSM* and *OSMR* expressions (defined as the lower quartile and upper quartile of expression, respectively) were assessed using a two-sided Mann–Whitney test.

Data visualization was performed in R (v.4.1.3) using the ggplot2 package (v.3.4.1).

### MIDAS GNN models

#### Multimodal biomedical data integration

We encapsulated our rich multimodal database within the Hetionet biomedical graph to support geometric deep learning GNN model development^21^ (Fig. 1a,b and Supplementary Table 1). Briefly, Hetionet represents biomedical information as a heterogeneous network compiled from 29 public datasets. This comprehensive network includes 47,031 nodes, classified into 11 distinct entities: genes, compounds, anatomy, diseases, symptoms, side effects, biological processes, cellular components, molecular functions, pathways and pharmacological classes. These entities are interconnected by 2,250,197 edges of 24 types, representing various relationships between the nodes. Gene–disease associations, GiG and GRN were extracted from the Hetionet biomedical knowledge graph^21^. Both networks are represented as unweighted networks, where an edge connects two gene nodes only if their products interact (GiG) or influence the expression of the target nodes (GRN), the latter also having directionality.

Graph nodes (*n* = 11,919) comprised genes with edges linking genes involved in *n* = 99,275 interactions (Fig. 1b and Supplementary Table 1). Genes were also labelled with *n* = 6,387 gene-autoimmune or rheumatic disease associations and their respective node degree within the GRN. To reduce input omics data dimensionality, bulk data were first pre-processed to describe the association between each data type and CPI response. Bulk transcriptomics and copy number data were represented as the median expression and log[R] values, respectively, across patient samples, categorized by response to CPI therapy. Mutation data were summarized as the mean weighted sum of mutation types (loss of function = 2, missense = 1, otherwise = 0) for R and NR patients.

The scRNA-seq atlas data, following the use of the SCTransform function, were pre-processed to create cell-type-specific interactomes that describe the role of different intratumoural immune subsets using the SCINET package (v.1.0)^93^. These interactome networks were subsequently summarized using the SCINET topological specificity score^93^. Briefly, this is a measure of gene influence within a specific cell-type interactome that accounts for its role in a global cell-type-agnostic reference. HLA-peptidomics, CRISPR co-culture screens and GWAS catalogue data were pre-processed as described above and appended to the node feature matrix. This effectively reduced data dimensionality and allowed the creation of meaningful features for integration along the GiG skeleton.

Missingness was classified into pseudo-missing and true-missing features. In pseudo-missing features, the missing values were imputed as 0: Shapley importance scores from CPI response predictors, genetic SNP–phenotype associations and GiG network degrees. In true-missing features, certain genes were absent and could not reliably be imputed with 0: gwCRISPR co-cultures (excluded missing genes), HLA-peptidomics (median imputation) and GRN node degrees (median imputation). HLA-peptidomics missing training data and held-out sets were imputed using the median value computed on the training set. Missing GRN degrees in the training data were imputed using the median value computed on the training set, and those in the held-out dataset were imputed using that computed on the held-out set. Missing gene topological specificity scores were imputed with 0.

#### Positive class labels for model development

For all the target discovery models, we defined an immuno-oncology target as genes that have been addressed in at least phase I clinical trials. Immuno-oncology targets (from 2017 to 2019) were downloaded from the Cancer Research Institute iAtlas (https://isb-cgc.shinyapps.io/iatlas/). After excluding targets identified by the terms ‘Pathway’ = ‘Antigen’ and ‘Description’ containing ‘vir’, ‘vaccine’, ‘cell therapy’ and ‘cellular therapy’, *n* = 332 true-positive immuno-oncology targets were obtained. After exclusions due to missing data, there were *n* = 260 positively and *n* = 11,659 negatively labelled genes for GNN development.

#### MIDAS GNN design space

We systematically studied the architectural design space for GNNs that could integrate rich multimodal data and leverage Hetionet for immuno-oncology target discovery^21^. Specifically, three key components were considered in the implemented approach^100^: (1) a stacked set of pre-processing MLP layers (allowing for feature engineering through combining the input features and the injection of nonlinearity), including dropout layers and batch normalization; (2) a module covering stacked GNN designs, for example, convolutional-based, attention-based, or sample and aggregate-based algorithm specifically designed for inductive node embedding that generalizes to unseen nodes^32,33,101,102^; and (3) a final post-processing module also comprising stacked MLPs (to refine the node embeddings and inject further nonlinearity, thereby increasing the overall network depth and expressiveness) and leading to a probability output^100^. We expanded the number of nodes from the pre-processing MLP module to the GNN (by a factor of 2) and contracted the GNN output to the post-processing MLP (by the same factor). We used a factor of 2 to reduce computational complexity that would otherwise arise from further optimizing this value. This design choice was inspired by similar approaches in CNNs (inverted bottleneck^103^) and transformers (feed-forward networks for expansion and compression^104^).

Hyperparameter search was done in Optuna (v.3.0)^105^ by maximizing the ROC-AUC in test folds during CV (see the ‘Resampling optimization strategies for target prediction models’ section) using the default tree-structured Parzen estimator. In addition to the message passing model-specific hyperparameters, the general hyperparameters optimized were embedding dimension, number of layers in the pre-processing and post-processing modules, learning rate and dropout rate (Supplementary Table 11). For each algorithm, the domains for hyperparameter search were meticulously crafted by extending the recommended search regions specified in the original papers of each algorithm.

#### MIDAS GIN architecture

The underlying graph for the GNNs was the GiG network extracted from Hetionet^21^. To evaluate the performance of various GNNs (Supplementary Methods describes their general structure), several architectures available through the PyTorch Geometric package (v.2.3.1)^106^ were used with an inductive learning paradigm. These comprised models such as graph sample and aggregate^32^, graph convolutional networks^102^, graph attention networks^101^ and variants of the GIN^33^. These variants comprised a GIN using MLPs (GIN MLP); a GIN MLP that did not implement any pre- or post-processing layers (GIN MLP no proc); a GIN MLP no proc variant that implemented global concatenation (GIN MLP no proc concat); and a GIN that uses a linear layer instead of an MLP, pre- and post-processing layers, and does not implement a global concatenation. The last variant had the highest performance and is referred to as MIDAS GIN. Therefore, the GIN message passing model and the characteristics of the optimal model are described below:

1 $${{\mathbf{x}}}_{\mathrm{i}}^{({\mathrm {k}})}={\mathrm{MLP}}((1+\epsilon )\cdot {{{\mathbf{x}}}_{\mathrm{ i}}}^{\left({\mathrm {k}}-1\right)}+\mathop{\sum }\limits_{\mathrm{j}\in {\mathscr{N}}\left({\mathrm {i}}\right)}{{{\mathbf{x}}}_{\mathrm{j}}}^{\left({\mathrm {k}}-1\right)}),$$

where ${{\mathbf{x}}}_{{\mathrm i}}^{({\mathrm k}-1)}$ and ${{\mathbf{x}}}_{{\mathrm j}}^{({\mathrm k}-1)}$ are the vector of features for each node *i* and all nodes *j* belonging to the neighbourhood of *i* (${\mathscr{N}}({i})$) in the (*k* – 1) stacked layer. The MLP is built from piling several modules of ${\rm{ReLU}}\left({\rm{BN}}\left({\rm{Linear}}\left(\,\right)\right)\right)$, where ${\rm{ReLU}}$ refers to a rectified linear unit, ${\rm{BN}}$ is a batch normalization layer and ${\rm{Linear}}$ denotes a linear layer. The output of equation (1) was then passed through a ${\rm{DROPOUT}}\left(\,\right)$ layer to avoid overfitting. This is close to the original architecture^33^. The parameter $\epsilon$ weighs the contribution of each node to its own embedding.

In our modified GIN (referred to as MIDAS GIN), we used a ${\mathrm{Linear}}\left(\,\right)$ layer instead of an MLP:

2 $${{\mathbf{x}}}_{\mathrm{ i}}^{({\mathrm {k}})}={\mathrm{DROPOUT}}({\mathrm{ReLU}}({\mathrm{BN}}({\mathrm{LINEAR}}(\left(1+\epsilon \right)\cdot {{{\mathbf{x}}}_{\mathrm{i}}}^{\left({\mathrm {k}}-1\right)}+\mathop{\sum }\limits_{\mathrm{j}\in {\mathscr{N}}\left({\mathrm {i}}\right)}{{{\mathbf{x}}}_{\mathrm{j}}}^{\left({\mathrm {k}}-1\right)})))),$$

The use of the dropout, rectified linear unit (ReLU) and batch normalization layers are justified in the Supplementary Methods. The sum was used in $\mathop{\sum }\limits_{\mathrm{j}\in {\mathscr{N}}\left({\mathrm {i}}\right)}{{{\mathbf{x}}}_{\mathrm{j}}}^{\left({\mathrm {k}}-1\right)}$ as this was proven to confer the largest expressivity to the GIN model^33^. As mentioned above, each of these message passing layers was combined with the pre-processing (equation (3)) and post-processing (equation (4)) layers:

3 $${{\mathbf{x}}}_{\mathrm{i}}^{\mathrm{m}}={\mathrm{DROPOUT}}^{\left({\mathrm {m}}-1\right)}({\mathrm{ReLU}}^{\left({\mathrm{m}}-1\right)}({\mathrm{BN}}^{\left({\mathrm{m}}-1\right)}({\mathrm{LINEAR}}^{\left({\mathrm{m}}-1\right)}({{\mathbf{x}}}_{\mathrm{i}}^{\left({\mathrm {m}}-1\right)})))),$$

4 $${{\mathbf{x}}}_{\mathrm{i}}^{\mathrm{n}}={\mathrm{DROPOUT}}^{\left({\mathrm {n}}-1\right)}({\mathrm{ReLU}}^{\left({\mathrm{n}}-1\right)}({\mathrm{BN}}^{\left({\mathrm{n}}-1\right)}({\mathrm{LINEAR}}^{\left({\mathrm{n}}-1\right)}({{\mathbf{x}}}_{\mathrm{i}}^{\left({\mathrm {n}}-1\right)})))),$$

where *m* and *n* represent the number of stacked layers for each module, that is, pre-processing (equation (3)) and post-processing (equation (4)), respectively.

All models were assessed using a rigorous CV resampling strategy, as detailed later.

To improve the generalization ability of the models to external datasets under the inductive paradigm and to enhance training speed, all models were optimized for the number of sampling *k*-nearest neighbours, with the search being over *k*-nearest neighbour values of 50, 100 and 200 (ref. ^32^). This affects the percentage of nodes in ${\mathscr{N}}{\mathscr{(}}i)$ that is taken into account during message passing. The top performance was achieved with the GIN model (*k*-nearest neighbours = 50) represented in equation (2) with pre-processing and post-processing layers (Supplementary Table 11 lists the optimal hyperparameters).

#### Interpretability of MIDAS graph models by permutation feature importance and degree-preserving graph null models

We quantified the importance of each feature using permutation analysis. The influence of a feature was assessed by examining the change in the prediction performance of the model, measured by the difference between the ROC-AUC on the CV training set and the mean ROC-AUC following 500 permutations of that feature. Each feature was permuted individually and then grouped into biological categories, meaning that some biological categories (which map to multiple features) will have >500 permutations for each fold. For data visualization purposes, the standard error was computed using the standard deviation across the differences between the original performance and that following each permutation. This provided a concise insight into the attributes of the model without necessitating further training.

We repeated this permutation analysis on the CV and held-out test sets to investigate the consistency across data splits. Here, the permutation feature importance scores are measured as the absolute difference in ROC-AUC between that computed on the original dataset and the mean across the corresponding permuted versions. Therefore, the original performance across the CV training folds (CV train performance) was compared against the performance across the permuted CV training folds, the original performance across the CV test folds (CV test performance) against the permuted CV test folds, and the original held-out test performance against the permuted version. As a further sensitivity analysis, we investigated how feature importances vary across specificity thresholds (0.7, 0.8 and 0.9). Model predictions were categorized to achieve these specificity values and the permutation feature importance was measured as the absolute change in recall post-permutation.

Additionally, we evaluated whether biological information encapsulated within the connectivity of the underlying network provided value to the model compared with a null distribution obtained by local edge swapping (500 times) whilst preserving the node degree distribution. Edge permutations were performed using the Python package Xswap (v.0.0.2 (ref. ^107^)), associated with the Hetionet project^21^. The permutation importance was defined as the difference between the ROC-AUC of the training set and that following degree-preserving edge permutation.

We also used an additional interpretability strategy to determine the contribution of specific pathways to the output scores of nodes identified as potential targets. The graph structure reflects gene–gene interactions; therefore, gene nodes are connected if their products interact with each other. Consequently, it is naturally organized into biological pathways (since proteins involved in the same pathway interact to propagate signals, leading to the functional effect of that pathway^108,109^). GNN message passing is constrained by these GiG edges and determines the model output. Therefore, information flow within the network occurs along interacting genes and, thus, the biological pathways to which they correspond.

Pathway permutation importance was calculated by permuting only edges that span gene nodes involved in a given pathway. To calculate pathway permutation importance for a specific predicted target, the target was first mapped to its manually curated pathways. For each pathway in turn, only the edges connecting genes belonging to that pathway were shuffled (in a degree-preserving manner), keeping all other edges constant. In this manner, only information flow within that specific biological pathway was corrupted, whereas flow through any other pathway remained unchanged.

This was repeated and the predicted score for the candidate target of interest was obtained for each of the 100°-preserving permutations for each pathway in which the target was involved. The importance of a pathway for target prediction was quantified as the median absolute normalized (as shown below) change in the model output score. Following the permutation of a given pathway, if there is a large change in the corresponding model prediction for the candidate target of interest, it suggests that this pathway highly influences the output prediction for that candidate target. On the other hand, if the output score was robust to the permutation of GiG edges within a specific pathway, it follows that this pathway is unlikely to be critical for predicting the candidate target of interest.

To account for the influence of pathway size on importance (Supplementary Fig. 4a), we normalized the results by the square root of the edge count (Supplementary Fig. 4b). This method assessed feature importance for the trained model and, therefore, did not retrain the model post-permutation. In this manner, the issue of redundant information contained in potentially collinear features is bypassed.

### Train–test split for target prediction models

For the prediction of immuno-oncology targets, a dataset comprising a total of 15,261 genes was stratified into distinct subsets for training and validation purposes. Specifically, 75% of the genes (*n* = 11,442) were randomly allocated to the training set in which CV was performed. The held-out dataset, which included the remainder of the genes (*n* = 3,819), was excluded from all training and optimization processes. This data split was stratified by the immuno-oncology target status and missingness for all input variables, utilizing the MultilabelStratifiedShuffleSplit() function from the iterative-stratification package (v.0.1.7)^110^ to ensure balanced representation.

Since not all genes were present in the GiG network, a total of *n* = 11,919/15,261 (78.1% of the maximum possible) genes in the integrated dataset were used to develop the MIDAS variants. Within this subset of genes present in the GiG network, the training set comprised *n* = 8,933/11,442 (78.1%), whereas the held-out test set contained *n* = 2,986/3,819 (78.2%).

### Resampling optimization strategies for target prediction models

Within each training fold, the allocation of genes to CV subsets was further stratified by the immuno-oncology target status. To address class imbalance, random undersampling of the majority class was implemented for each training fold using techniques from the imbalanced-learn package. Hyperparameter optimization for the ensemble meta-learners and GNN models was conducted under a robust framework of ten-times-repeated tenfold CV. Consistency was maintained across the meta-learner (Supplementary Methods) and GNN techniques by using the same random seed in the samplers, thereby facilitating a fair comparison of their performances.

For consistency, the ROC-AUC metric was used to evaluate model performance across validation folds and in the held-out set, as well as to compare against existing target discovery methods. Since the output probability scores were not categorized into discrete predicted labels (which would require optimizing the threshold value), metrics requiring distinct categorical class label predictions would be less suited to capturing model behaviour. Furthermore, the ROC-AUC measure is a standard metric in binary classification, including for approaches that do not rely on GNNs or deep learning^5,22,70,111,112^. Additionally, it is independent of prevalence, unlike other metrics such as Matthew’s correlation coefficient and the precision–recall curve. Finally, we randomly undersample the majority set to balance the classes, justifying the use of the ROC-AUC.

Immuno-oncology candidate targets were subsequently ranked based on their probability of being a target, as predicted by both meta-learner (Supplementary Methods) and GNN models. Each model derived from the CV training folds was utilized to propose candidates from both the corresponding CV test folds and held-out dataset. This process generated probability distributions for each gene, from which the mean score was calculated (that is, bagging across CV folds) and used to rank the candidates. Therefore, individual predictions for a specific gene come only from CV fold models in which this gene was not included in any CV training folds. This methodology ensured a comprehensive evaluation and ranking of potential immuno-oncology targets based on their predicted likelihood of being true targets.

### Benchmarking against alternative methods

We benchmarked our proposed immuno-oncology target discovery system against multiple alternative methods using the held-out test set. OpenTargets (https://www.opentargets.org/) scores were filtered to those for the indication ‘cancer’ and downloaded. Both direct evidence scores (which directly links a gene to a disease) and indirect evidence scores (leveraging disease hierarchical classifications to postulate target–disease links) were separately tested, as was a combination method that aggregated both scores by the median value. TargetDB is an RF model that predicts the tractability probability for all genes^34^. It has an MPO tool for differentially weighting evidence sources (for example, structural data or genetic links). Both tractability estimates and MPO scores (with equal weights) were tested. DepMap (https://depmap.org/portal/) gene effect scores (which reflect the influence of gene knockout/knockdown on cell line viability) were downloaded for genes within the held-out test set and assessed for the ability to identify known immuno-oncology targets. We removed data for all carcinomas in situ or non-solid tumours, as our focus with MIDAS was to identify immunotherapy drug targets for established solid tumours. Finally, MAGeCK scores from the CRISPR cancer–CD8^+^ T cells co-cultures were investigated for immuno-oncology target identification. These ability of these alternative methods to identify immunotherapy drug targets was assessed, by the ROC-AUC, in a binary immuno-oncology target classification task.

### In silico model validation

To robustly validate the target discovery models, we devised multiple external and orthogonal in silico validation exercises (Fig. 1c). First, we assessed model generalization by investigating predictions for genes that had entered stage I immuno-oncology clinical trials after we froze our dataset for model development in 2019. ClinicalTrials.gov (https://clinicaltrials.gov/) was searched (in November 2023) using the following parameters: condition/disease = cancer; intervention/treatment = immunotherapy; sex = all; age = all; study phase = early phase I or phase I; study type = interventional; earliest date: 01/01/2020. This retrieved *n* = 453 results.

We extracted *n* = 48 new, time-sliced targets (*n* = 36 in the graph system after accounting for gene exclusions due to missingness) that were not annotated as such during model training (Fig. 1c). MIDAS predictions for these time-sliced targets were compared with a null distribution generated by the mean prediction across 1,000 size-matched randomly sampled gene sets. To avoid artificially decreasing the values in the null distribution, we did not exclude the time-sliced targets when performing the random sampling. Empirical *P* values were used to assess statistical significance.

Next, we examined the model using orthogonal tasks. The first such task was whether models could discriminate targets that were clinically approved from those undergoing clinical trials (Fig. 1c). We extracted clinical phase information for targets from the Cancer Research Institute (https://isb-cgc.shinyapps.io/iatlas/), aggregating by the maximum phase for all trials addressing the same target. We did not include the time-sliced targets mentioned above in this analysis. Model predictions for targets in each group were then compared using the Kruskal–Wallis and Mann–Whitney *U* tests.

We then investigated whether our modelling systems could identify genes that were differentially expressed between patients who did and did not respond to CPI therapy (Fig. 1c). We used unseen bulk RNA count data from the CPI2500 cohort (an extension of our previous CPI1000+ cohort^3^), comprising *n* = 658 new patients (*n* = 129 had disease that responded to CPI, whereas *n* = 529 had disease that did not) from three studies that were not used during the development of either model.

DEGs were identified for pan-, lung (*n* = 59 R and *n* = 321 NR), bladder (*n* = 34 R and *n* = 108 NR) and renal (*n* = 36 R and *n* = 100 NR) cancers using the DESeq2 (v.1.34.0) R package^113^, adjusting for the effects of study and patient sex. All bladder cancer samples originated from the same study and, therefore, this variable was dropped from the DESeq2 analysis. For the pan-cancer setting, we further adjusted for tumour type. The intersections between these DEGs and the top 200 predicted targets from MIDAS GIN were computed and compared with that generated from a null distribution of 1,000 randomly sampled (stratified by expression), size-matched gene sets. The Spearman correlation between MIDAS predictions and the Wald test statistic for the top 1,000-ranked genes was also assessed in bins of *n* = 200 genes.

### Candidate target triage

The top 300-ranked predictions were manually reviewed to exclude genes labelled as known immuno-oncology targets for training (excluding *n* = 101), as well as targets with obvious immune functions or targets unlikely to be relevant to immuno-oncology, to prioritize *n* = 43 potentially novel immunotherapy targets. The exclusion of highly obvious immune targets was confirmed through comparing the significant biological processes that were overrepresented between the initial ranked list and the resulting shortlist, observing that the vast majority of immune pathways were depleted (Supplementary Fig. 10).

From this shortlist, targets were excluded if they were a named target of a clinical candidate drug at phase I or above in an oncology indication (Cortellis, https://access.clarivate.com/login?app=cortellis; OpenTargets, https://www.opentargets.org/), were common essential genes or were not expressed in immune cells (Human Protein Atlas, https://www.proteinatlas.org/).

Targets were prioritized for literature review if immune cell expression was high globally or differentially in subsets. Reviewed targets were prioritized if there was literature supporting a plausible link to an immuno-oncology mechanism (PubMed, https://pubmed.ncbi.nlm.nih.gov/), and deprioritized if existing structure and druggability scores indicated a difficult-to-drug target (TargetDB^34^, Cansar, https://cansar.ai/). Target selection for the ex vivo assays required tool compounds to be commercially available.

### Functional validation of candidate immunotherapy targets

#### Study ethics and research compliances

The study involved materials from TRAcking Cancer Evolution through therapy (Rx) (TRACERx) melanoma: exploratory analysis of genomic signatures of progression in melanoma (TRACERx Melanoma) (IRAS:68421). The study was reviewed and approved by both the Royal Marsden Committee for Clinical Research (CCR) (CCR:3569) and the London-Chelsea Research Ethics Committee (REC) (REC: 11/LO/0003), and was performed in compliance with all relevant ethical regulations.

#### PDEs

##### Patient characteristics, patient-derived tumour material procurement, processing and cryopreservation

All patients provided written consent for tissue samples that were not required for diagnosis to be used for research purposes. Patient-derived materials were collected from patients diagnosed with melanoma at the Royal Marsden Hospital. Patient characteristics are described in Supplementary Tables 9 and 10.

Tumour tissues were obtained from surgical resections and were macroscopically selected by a pathologist. Parts of the tumour were collected in an ice-cold collection medium (University of Wisconsin Solution (UW Solution, Bridge to Life) supplemented with 100 μg ml^−1^ of Primocin (InvivoGen)) for subsequent tumour processing and cryopreservation. Tumour materials were immediately processed by manual sectioning into small fragments of 1–2-mm^3^ size on a CoolBox XT Workstation (Corning).

After processing, fragments from different spatial regions were mixed together (to minimize heterogeneity across PDEs derived from the same patient sample) and were frozen in cryovials containing 1 ml of 90% fetal bovine serum (FBS; Gibco) and 10% dimethyl sulfoxide (DMSO; Sigma-Aldrich) with 15 fragments per vial. All samples were cryopreserved in liquid-phase nitrogen until future usage.

##### Human PDE cultures

For each well of a 96-well plate, a single tumour fragment was embedded in the extracellular matrix (ECM) containing sodium bicarbonate (7.5% (Gibco)), Collagen I (final concentration, 1 mg ml^−1^ (Corning)), Matrigel (final concentration, 4 mg ml^−1^ (Corning)) and tumour medium (Dulbecco’s modified Eagle’s medium (Gibco) supplemented with 1 mM of sodium pyruvate (Sigma-Aldrich), 1× Minimum Essential Medium non-essential amino acids (Sigma-Aldrich), 1× GlutaMax (Gibco), 10% FBS and 1% penicillin–streptomycin). The ECM was prepared on ice by the slow mixing of the components in the order listed above. To each well of the plate, 40 µl of ECM was added and the plate transferred to a 37 °C incubator for ≥30 min to solidify.

To thaw the cryopreserved PDEs, vials were thawed in a 37 °C water bath until only a small amount of ice remained. Tumour fragments were then transferred to a 50 ml centrifuge tube and a prewarmed wash medium (Dulbecco’s modified Eagle’s medium (Gibco), 10% FBS and 1% penicillin–streptomycin) was slowly added up to 10 ml. The tumour fragments were then washed by transferring to a cell strainer and sequentially lowering the contained fragments into three wells of a six-well plate each containing 7 ml of wash medium.

After thawing and washing, one fragment was placed on top of the solidified ECM in each well and an additional 40 µl of the ECM was added on top, and then incubated for at least 30 min before subsequent treatment. After ECM solidification, 120 µl of the tumour medium was added to each well on top of the ECM.

For untreated wells, the tumour medium was supplemented with DMSO (1:1,000, Sigma-Aldrich) as a negative control for all experiments. For treated wells, the medium was supplemented with the PTPN22 inhibitor (11 µM, MedChem Express) or anti-OSM antibody (5 µg ml^−1^, Bio-Techne) to perturb PTPN22 and OSM-OSMR, respectively.

##### Flow cytometry analysis of PDEs

For the analysis of T cell phenotype and activation states (Fig. 5a), PDEs were analysed by high-dimensional flow cytometry after culture using the following antibodies: BUV395 Mouse Anti-Human Ki-67 (RRID: AB_2738577, Clone: B56, BD Biosciences, 1:40), BUV496 Mouse Anti-Human CD8 (RRID: AB_2870223, Clone RPA-T8, BD Biosciences, 1:80), BUV563 Mouse Anti-Human CD45RA (RRID: AB_2870211, Clone: HI100, BD Biosciences, 1:80), BUV737 Mouse Anti-Human CD39 (RRID: AB_2738919, Clone: TU66, BD Biosciences, 1:20), BUV805 Mouse Anti-Human CD3 (RRID: AB_2870181, Clone: SK7, BD Biosciences, 1:40), BV480 Mouse Anti-Human CD103 (RRID: AB_2743774, Clone: Ber-ACT8, BD Biosciences, 1:40), BV711 Mouse Anti-Human HLA-DR (RRID: AB_2738378, Clone: G46-6, BD Biosciences, 1:40), BB790-P Anti-Human CD4 (RRID: N/A, Clone: SK3, BD Custom Conjugates, 1:160), Alexa Fluor 700 Mouse Anti-Human GZMB (RRID AB_1645453, Clone: GB11, BD Biosciences, 1:80), Brilliant Violet 421 Mouse Anti-Human CD279 (PD-1) (RRID: AB_10960742, Clone: SK3, BioLegend, 1:20), Brilliant Violet 650 Mouse Anti-Human CD197 (CCR7) (RRID: AB_2563867, Clone: G043H7, BioLegend, 1:10), Brilliant Violet 785 Mouse Anti-Human CD45 (RRID: AB_2563129, Clone: HI30, BioLegend, 1:20), FITC Anti-Human HLA-A, HLA-B, HLA-C (RRID: AB_314873, Clone: W6/32, BioLegend, 1:40), PE Mouse Anti-Human TCF1 (TCF-7) Antibody (RRID: AB_2728492, Clone: 7F11A10, BioLegend, 1:10), PE/Dazzle 594 Mouse Anti-Human CD137 (4-1BB) (RRID: AB_2566260, Clone: 4B4-1, BioLegend, 1:20), PE/Cyanine7 Mouse Anti-Human CD134 (OX40) (RRID: AB_10901161, Clone: Ber-ACT35, BioLegend, 1:20), APC Rat Anti-Human Foxp3 (RRID: AB_1603280, Clone: PCH101, Invitrogen, 1:40).

For the analysis of treatment effects, PDEs were manually retrieved from the ECM after 48 h of culturing. Tumour fragments were pooled for each condition and processed into single-cell suspensions by enzymatic digestion on a rotator at 37 °C for 45 min with a digestion mixture (RPMI 1640 supplemented with 1 mg ml^−1^ of collagenase type IV (Sigma-Aldrich) and 25.2 µg ml^−1^ of DNAse I (Sigma-Aldrich)). Samples were subsequently washed with ice-cold PBS, followed by manual mashing through a 100-µm filter (Miltenyi Biotec).

For flow cytometry staining, cells were Fc-blocked with Human TruStain FcX Fc Receptor Blocking Solution (BioLegend) for 20 min at room temperature together with chemokine receptor staining (Brilliant Violet 650 Mouse Anti-Human CD197 (CCR7)). Antibodies for surface markers (BUV496 Mouse Anti-Human CD8, BUV563 Mouse Anti-Human CD45RA, BUV737 Mouse Anti-Human CD39, BUV805 Mouse Anti-Human CD3, BV480 Mouse Anti-Human CD103, BV711 Mouse Anti-Human HLA-DR, BB790-P Anti-Human CD4, BV785 Mouse Anti-Human CD45, BV421 Mouse Anti-Human CD279 (PD-1), FITC Anti-Human HLA-A, HLA-B, HLA-C, PE/Dazzle 594 Mouse Anti-Human CD137 (4-1BB), PE/Cyanine7 Mouse Anti-Human CD134 (OX40) and eBioscience Fixable Viability Dye eFluor 780) were prepared in a mixture of Brilliant Staining Buffer Plus (BD Biosciences) and FACS buffer (PBS containing 2% FBS and 5 mM of EDTA). Blocked cells were stained in the antibody mix for 30 min at 4 °C.

For intracellular staining, the cells were washed three times and then fixed and permeabilized using the FOXP3 Transcription Factor Staining Buffer set (Thermo Fisher Scientific) at room temperature in the dark for 30 min. Cells were again washed three times and then resuspended in 50 µl of staining mix containing antibodies for intracellular markers (BUV395 Mouse Anti-Human Ki-67, BUV496 Mouse Anti-Human CD8, BUV805 Mouse Anti-Human CD3, BB790-P Anti-Human CD4, Alexa Fluor 700 Mouse Anti-Human GZMB, PE Mouse Anti-Human TCF1 (TCF-7) and APC Rat Anti-Human Foxp3 antibodies) and incubated at room temperature for 30 min. The samples were washed three times before acquisition.

Samples were acquired on a BD FACSymphony A5 (BD Biosciences) and data collected using the BD FACS Diva software v.9.1 (BD Biosciences)^114^, with further analysis performed using FlowJo (v.10.9.0), Prism (v.10.0.2; GraphPad) and R (v.4.1.3). All data were analysed using paired two-sided Wilcoxon signed rank tests. An example of the gating strategy is shown in Supplementary Fig. 11.

##### Cytokine and chemokine analysis

For the analysis of chemokines and cytokines, the explant culture supernatants were collected after 48 h of culturing. The supernatants from each condition were pooled and centrifuged at 450*g* for 5 min twice to remove any debris. Supernatants were immediately frozen and stored at −80 °C until future usage. The concentration of the analytes was quantified using LEGENDplex cytometric bead array. Supernatants were thawed on ice, and the presence of the indicated cytokines and chemokines was detected using the LEGENDplex 14-plex customized Human Proinflammatory Chemokine and Human CD8/NK panels (BioLegend). The assay was performed, and the samples were acquired on the Novocyte Quanteon flow cytometer (Agilent Technologies) according to the manufacturer’s instructions. The concentrations of the indicated analytes were quantified using LEGENDplex cloud-based data analysis. All data were analysed using paired two-sided Wilcoxon signed rank tests in R (v.4.1.3).

### Reporting summary

Further information on research design is available in the Nature Portfolio Reporting Summary linked to this article.

## Supplementary information

Supplementary Information Supplementary Note 1, Tables 1–11, Figs. 1–11 and Methods.

Reporting Summary

Supplementary Tables Supplementary Tables 1,6,7,10.

Supplementary Data Supplementary Data 1–10.

## Source data

Source Data Fig. 2 Statistical source data.

Source Data Fig. 3 Statistical source data.

Source Data Fig. 4 Statistical source data.

Source Data Fig. 5 Statistical source data.

## Data Availability

The following datasets are publicly available: single-cell transcriptomics (Methods provide full details on individual studies), HLA-peptidomics^28^, CRISPR co-cultures^18,19^, GWAS catalogue^20^ and Hetionet^21^. Node permutation data for Fig. 3 are available via GitHub at http://github.com/augustgm/MIDAS. Bulk sequencing patient cohorts are described elsewhere: CPI1000+ (ref. ^3^) and CPI2500 (paper in preparation). Further information and requests for resources should be directed to C.S. PDEs were derived from metastatic melanoma samples obtained in the TRACERx Melanoma trial. Due to patient confidentiality and sample limitation restrictions, these biological materials are not available. Source data are provided with this paper.

## Code availability

The source code to train the MIDAS GIN model and perform interpretability analyses using a demonstration dataset is available via GitHub at https://github.com/augustgm/MIDAS (ref. ^115^). The repository also contains code to recreate all the main figures and is publicly available as of the date of publication.
