# Supplementary material for: Immunotherapy drug target identification using machine learning and patient-derived tumour explant validation
Source: Nat Mach Intell. 2026 May 18;8(5):670–89. doi: 10.1038/s42256-026-01201-3 (PMC13201160; doi:10.1038/s42256-026-01201-3)
Supplement: Supplementary file 1 — Supplementary Note 1, Tables 1–11, Figs. 1–11 and Methods. [file 42256_2026_1201_MOESM1_ESM.pdf]

nature machine intelligence

Supplementary information https://doi.org/10.1038/s42256-026-01201-3

Immunotherapy drug target identification using machine learning and patient-derived tumour explant validation

In the format provided by the authors and unedited

Table of Contents

Supplementary Note 1 2

Pathways enriched in MIDAS GIN predictions 2

Supplementary Methods 2

Check-point inhibitor (CPI) response prediction 2

Meta-learning approach for immuno-oncology target discovery 4

General graph neural network architecture 5

Benchmarking against standard deep neural networks 6

Supplementary Tables 8

Supplementary Table 1: Node features for MIDAS GNN immuno-oncology targets prediction models 8

Supplementary Table 2: Performance of GNN variants 8

Supplementary Table 3: Top 15 predictions from MIDAS GIN 9

Supplementary Table 4: Performance of linear, tree-based, and MLP baselines. 10

Supplementary Table 5: Optimal hyperparameters for linear, tree-based, and MLP baselines .. 10

Supplementary Table 6: Pathways enriched within MIDAS GIN predictions 13

Supplementary Table 7: Permutation feature importance across specificity thresholds 13

Supplementary Table 8: Shortlisted candidate targets 13

Supplementary Table 9: Summary patient clinical characteristics 14

Supplementary Table 10: Individual patient clinical characteristics 14

Supplementary Table 11: MIDAS GIN optimal hyperparameters 15

Supplementary Figures 16

Supplementary Figure 1: Performance of different GNN models at identifying known immuno-oncology targets in clinical development 16

Supplementary Figure 2: Meta-learning framework for immuno-oncology target discovery 18

Supplementary Figure 3: Node feature permutation importance across train-validation splits 19

Supplementary Figure 4: Pathway permutation approach 20

Supplementary figure 5: OSM expression is associated with altered T cell scores by CIBERSORT analysis of bulk transcriptomics data 21

Supplementary figure 6: OSMR expression is associated with altered T cell scores by CIBERSORT analysis of bulk transcriptomics data 22

Supplementary figure 7: OSMR expression is associated with altered macrophage cell scores by CIBERSORT analysis of bulk transcriptomics data 23

Supplementary figure 8: OSM expression is associated with altered macrophage cell scores by CIBERSORT analysis of bulk transcriptomics data 24

Supplementary Figure 9: Validation of genes with high topological specificity scores in scRNA-seq data 25
Supplementary Figure 10: Comparison of overrepresented pathways amongst the top 300 ranked genes and the shortlisted candidates 26
Supplementary Figure 11: Representative gating strategy for PDE assays 27
References 28

Supplementary Note 1

Pathways enriched in MIDAS GIN predictions

GSEA revealed that MIDAS GIN predictions enriched for multiple immune system pathways (Fig. 3a). IL-10 is a potent anti-inflammatory cytokine that is produced by both myeloid and lymphoid cells. It acts on macrophages to inhibit pro-inflammatory cytokine release and reduce MHC-II expression, thus interfering with antigen presentation. IL-2 is a T cell cytokine that promotes T cell proliferation and differentiation as well as Treg maintenance and function. IL-6 is a pro-inflammatory cytokine produced by macrophages and lymphocytes that is involved in the acute phase response alongside lymphocyte activation, differentiation, and antibody production. Strikingly, mRNA 3'-end processing has a negative normalised enrichment score (NES), which could reflect reduced activity of the corresponding processing unit due to the shortened 3' untranslated regions (UTR) observed in cancer cells (Mayr & Bartel, 2009).

Supplementary Methods

Check-point inhibitor (CPI) response prediction

Models for Bulk transcriptomics and exome data

Models predicting CPI response were created using extreme gradient boosting techniques available from the XGBoost Python package (Chen & Guestrin, 2016) (version 2.1.0). We employed nested cross-validation (CV) with 10 train-test divisions (75% train, 25% test) to verify the consistency of prediction performance. Within each training set, model hyperparameters were optimised, in Optuna (Akiba et al., 2019), using ten-times-repeated tenfold CV, with ROC-AUC as the metric and sample allocation to folds stratified by response status and cancer type. The default Optuna tree-structured Parzen estimator was employed for this purpose. The domains for hyperparameter search were those recommended in the XGBoost package (Chen & Guestrin, 2016). Random undersampling of the majority class was implemented for each training fold using the imbalanced-learn package (Lemaitre

et al., 2017) at a ratio of 1 to 1. The optimal hyperparameters were then used to fit an XGBoost regularised classifier on the entirety of each training set with the performance evaluated on the corresponding test set. Feature importance was determined according to SHAP (Shapley Additive exPlanations) Python package (version 0.45; (Lundberg et al., 2017)) and global absolute average values were aggregated across training sets.

Mutation-associated neoantigen scores

Most single-cell transcriptomics (scRNA-seq) datasets lacked immunotherapy response labels. Therefore, a previously published measure, the mutation-associated neoantigen (MANA) score, was utilised as a surrogate label. This MANA score comprises a gene signature that was calculated for all $CD8^+$ T cells (CD8A expression > 0) using AddModuleScore() from the Seurat package v4 (Hao et al., 2021). We aggregated these scores for each sample by the upper quartile value to yield sample-specific representations of tumour microenvironment (TME) immune dynamics. Different MANA score definitions exist for skin and non-skin tumours (Caushi et al., 2021; Oliveira et al., 2021); thus, we used the appropriate gene signature for each of these categories. Since neoantigen-specific T cells are important mediators of CPI-unleashed anti-tumour immune responses (Fehlings et al., 2017, 2019; George et al., 2017; Kamphorst et al., 2017; Rizvi et al., 2015; Van Rooij et al., 2013), this metric serves as a valid surrogate of clinical response. However, limitations include that clinical response depends on other host- and tumour-specific factors (Litchfield et al., 2021), alongside diverse TME cell types (Pittet et al., 2022).

Single-cell RNA-seq data models

Raw count data were extracted for each cell subtype after excluding normal, dying, or unknown cell subtypes, as well as $CD8^+$ T subtypes (due to the risk of confounding, as the MANA score is defined on these subtypes). Following the exclusion of mitochondrial genes, these data were pseudobulked and normalised using the variance-stabilisation transformation from DESeq2 (version 1.34.0; (Love et al., 2014)). Principal components analysis using the top 500 most varying genes was performed to identify drivers of batch effects. The removeBatchEffect() function from the limma package (version 3.50.3) was used to regress out the effect of source study, mitochondrial DNA percentage, and the number of expressed genes with raw counts > 5. Successful batch correction was confirmed by inspecting PCA biplots constructed from the corrected data.

Elastic net regression models were selected due to their demonstrated ability to handle high-dimensional data and feature correlation, as well as model therapy responses in cancer settings.

(Mehmood et al., 2024). They were trained to predict sample-level MANA scores from pseudobulked, batch-corrected scRNA-seq data for each cell subtype using scikit-learn (version 1.0.1; (Pedregosa et al., 2011), with acceleration through scikit-learn-intelex 2021.4.0 (https://pypi.org/project/scikit-learn-intelex/)). Ten train-test splits (75% train) were created by stratifying on the MANA score decile. For each of these training sets, we performed hyperparameter optimization using Optuna and five-fold quintuple CV (stratifying these splits by MANA score deciles). The optimal hyperparameters were then used to fit an elastic net regularised linear regressor on the entirety of each training set, with the performance evaluated on the corresponding test set. To further characterise model behaviour, we additionally developed 100 randomised null models for each cell subtype by randomly shuffling sample MANA scores and repeating the modelling process for 100 randomised train-test splits (75% train). Model performances and feature importances were benchmarked against these randomised nulls. Feature importance was determined according to SHAP (SHapley Additive exPlanations) Python package (version 0.41; (Lundberg et al., 2017)) and global absolute average values were aggregated across training sets.

Meta-learning approach for immuno-oncology target discovery

Feature matrix including CPI prediction importance per gene

Feature importances associated with CPI prediction (excluding the copy number models due to low test set performances) and MANA regression models (excluding any models with mean square error (MSE) greater than two standard deviations above the median), for bulk and single cell data, respectively, were considered as potential predictors. The importances were generated using the SHAP package (Lundberg et al., 2017). To capture how antigen processing affects immunotherapy response, we downloaded publicly available HLA-peptidomic and matched bulk transcriptomic data for n = 74 samples, including n = 60 diagnosed with cancer (Bulik-Sullivan et al., 2018) and computed Pearson correlation coefficients for gene expression-HLA peptide presentation associations. Causal data on immune responses to genetic perturbation stemmed from n = 7 publicly available CRISPR tumour-T cell co-cultures and n = 15,442 SNP-phenotype links. Biological context was provided through node degrees from the gene-interaction-gene (GiG) and gene-regulates-gene (GrG) Hetionet networks. Imputations were performed as described in the Methods for features common to both approaches. The GiG network degrees (here included as a feature, whilst it formed the network topology for the MIDAS GIN) and the Shapley importance scores from CPI response and MANA score predictors were imputed as 0.

Meta-learners and Ensemble Stacking

Several meta-learners were trained on the feature matrix described above: elastic net (Pedregosa et al., 2011), XGBoost (Chen & Guestrin, 2016), random forest (Pedregosa et al., 2011) and support vector machine (Pedregosa et al., 2011). The probability output of each of the meta-learners was used to train a simple logistic regression stacker to predict immuno-oncology drug targets from the joint meta-learner output probability matrix (Fig. S2). This third layer permits the calculation of a weighted mean of the meta-learner output scores, thus leveraging the heterogeneity within these output scores to increase the robustness of predictions in external datasets (Whalen & Pandey, 2013).

General graph neural network architecture

Consider a graph G = (V, E), where the edges (E) and vertices (V) define an adjacency matrix (A) which, in this context, is derived from the GiG network extracted from Hetionet, as previously described (Himmelstein and Baranzini, 2015). The design and study of GNN layers is a vast area within deep learning, presenting considerable complexity and numerous challenges. However, the majority of layer types can be encapsulated within the framework of the message passing paradigm, which is succinctly represented by the following equation:

$$\mathbf{x}_{\mathrm{i}}^{\mathrm{k}} = \gamma^{(\mathrm{k})}\left(\mathbf{x}_{\mathrm{i}}^{(\mathrm{k}-1)}, \bigoplus_{\mathrm{j}\in\mathcal{N}(\mathrm{i})} \phi^{(\mathrm{k})}\left(\mathbf{x}_{\mathrm{i}}^{(\mathrm{k}-1)}, \mathbf{x}_{\mathrm{j}}^{(\mathrm{k}-1)}\right)\right) \qquad \textbf{Eq1}$$

In **Eq 1**, $\mathbf{x}_{\mathrm{i}}^{(\mathrm{k}-1)}$ and $\mathbf{x}_{\mathrm{j}}^{(\mathrm{k}-1)}$ are the vector of features for each node i and all nodes j belonging to the neighbourhood of i, $\mathcal{N}(\mathrm{i})$, in the (k-1)th stacked layer. $\bigoplus$ represents a permutation-invariant aggregation function (e.g. sum, mean, or max). In our implementation, these aggregations are provided by PyTorch Geometric and differentiated via the automatic differentiation engine implemented in PyTorch. Additionally, γ and ϕ denote differentiable functions, e.g. MLP, specific to each GNN layer type. This paradigm fundamentally describes the process by which node representations are iteratively updated through the aggregation and transformation of information from neighbouring nodes. The message passing paradigm thus serves as a unifying foundation for understanding and developing a wide array of GNN architectures (Bronstein et al., 2021).

The overarching architecture of the GNN classifiers was meticulously designed by integrating MLP pre-processing layers, several GNN layers, and post-processing MLP layers (You, Ying and Leskovec, 2020). Optimisation efforts concentrated on determining the optimal number of layers per module, along

with other critical hyperparameters. To enhance the stability of the output and mitigate overfitting, batch normalisation (BN) layers and dropout layers were incorporated into the model architecture. This comprehensive approach ensured robust performance and generalisation of the GNN models. The general structure is as follows:

$$\mathbf{x}_i^m = \mathrm{DROPOUT}^{(m-1)}(\mathrm{ReLU}^{(m-1)}(\mathrm{BN}^{(m-1)}(\mathrm{LINEAR}^{(m-1)}(\mathbf{x}_i^{(m-1)})))) \quad \textbf{Eq2}$$

$$\mathbf{x}_i^k = \mathrm{DROPOUT}^{(k-1)}(\mathrm{ReLU}^{(k-1)}(\mathrm{BN}^{(k-1)}(\gamma^{(k)}(\mathbf{x}_i^{(k-1)}, \bigoplus_{j\in\mathcal{N}(i)} \phi^{(k)}(\mathbf{x}_i^{(k-1)}, \mathbf{x}_j^{(k-1)}))))) \quad \textbf{Eq3}$$

$$\mathbf{x}_i^n = \mathrm{DROPOUT}^{(n-1)}(\mathrm{ReLU}^{(n-1)}(\mathrm{BN}^{(n-1)}(\mathrm{LINEAR}^{(n-1)}(\mathbf{x}_i^{(n-1)})))) \quad \textbf{Eq4}$$

Where m, n and k represent the number of stacked layers for each of the modules, i.e. pre-processing (Eq2), message passing (Eq3) and post-processing (Eq4), respectively.

The rectified linear unit (ReLU) is widely used in neural networks, including GNNs, because it has proven to be stable and perform well across many data types. Its simplicity and effectiveness make it a go-to activation function in many architectures, including models like EMOGI (Schulte-Sasse et al., 2021) and MOGONET (Wang et al., 2021), where ReLU and its leaky version, respectively, are used to maintain stability and prevent saturation. Optimising the transfer function beyond ReLU, such as using sigmoid or tanh, would add unnecessary computational burden whilst being unlikely to provide significant improvements. ReLU was also a key choice in the original GIN paper (Xu et al., 2019), further highlighting its reliability in graph-based tasks. Its ability to mitigate the vanishing gradient problem, encourage sparsity, and handle non-linear relationships in a computationally efficient manner makes it a far simpler and safer option compared to other activation functions, potentially leading to faster training and effective generalisation of GNNs.

Benchmarking against standard deep neural networks

Despite the transformative successes of deep neural networks in domains such as computer vision and natural language processing, their application to tabular data, a modality prevalent in critical sectors like healthcare, has been met with limited success. Gradient-boosted decision tree ensembles, such as XGBoost, consistently represent the state-of-the-art for tabular data, owing to their robustness, intrinsic handling of heterogeneous and incomplete features, and minimal preprocessing demands. In contrast, deep learning models typically necessitate extensive feature engineering, bespoke

architectures, and exhaustive hyperparameter optimisation, often underperforming XGBoost baselines, particularly in data-limited regimes where their high parametric capacity cannot be sufficiently constrained (Shwartz-Ziv & Armon, 2022). Recent work has found that gradient-boosted trees still outperformed off-the-shelf neural network methods in tuning efficiency and computational cost, except when deep models were extensively ensembled alongside trees (Shwartz-Ziv & Armon, 2022).

To rigorously evaluate the advantage conferred by inductive graph neural networks (GNNs) operating on domain-specific biological structures, we also benchmarked our proposed models against linear models (elastic net regularised logistic regression), tree-based ensembles (XGBoost), and bespoke multilayer perceptron (MLP) models (see below). This comparison was conducted under strictly controlled conditions: models were trained from scratch, with the same hyperparameter optimiser (see main text) and CV splits as employed for the GNN (see Fig. 1a-b). These alternative, non-GNN models were trained on the same feature matrix as the GNNs, with the exception that the GiG network degree centrality was used as a feature (since these models do not operate on the GiG network topology directly, unlike the GNNs). This experimental design ensures that any observed performance difference can be attributed to the architectural paradigm, specifically, the efficacy of message passing on a curated knowledge graph versus a data-driven feature selection approach. These results complement our primary investigations, which benchmark our GNN-based method against both standard machine learning algorithms and established target identification models (section "Graph ML system achieves robust performance across *in silico* immuno-oncology benchmarks"; Figs. 2 and S1).

Two MLP configurations were evaluated. In the first, we applied the graph neighbour sampler (k-nearest neighbours (KNN) = 50) utilised by the full GNN models described in the main text (Hamilton et al., 2017). Integrating this sampler into our CV procedure helped convert the GNN framework into an inductive setting, thereby enhancing model generalisability. By employing the same sampling strategy, this MLP variant inherits some structural information from the knowledge graph, effectively mirroring the initial MLP pre-processing layer of the full GNN model (see Eqs 2 to 4 in the main text). The second MLP variant omitted the graph neighbour sampler entirely, thus removing any relational information provided by the knowledge graph and leveraged by the full GNN model.

The performances of these linear, tree-based, and MLP baselines are provided in Supplementary Table 4. Optimal hyperparameters for these models are provided in Supplementary Table 5.

Supplementary Tables

Supplementary Table 1: Node features for MIDAS GNN immuno-oncology targets prediction models

Attached as a separate Excel file.

Supplementary Table 2: Performance of GNN variants

GNN	KNN	AUC	Lower CI	Upper CI	Set
GCN	50	0.989492	0.98211	0.996874	Train
GCN	50	0.837878	0.807167	0.868589	Test
GCN	50	0.868837	0.824879	0.912794	Held-out test
GCN	100	0.98851	0.982088	0.994932	Train
GCN	100	0.827881	0.794108	0.861653	Test
GCN	100	0.864056	0.82001	0.908102	Held-out test
GCN	200	0.999511	0.999087	0.999935	Train
GCN	200	0.847879	0.819915	0.875844	Test
GCN	200	0.860594	0.814483	0.906705	Held-out test
SAGE	50	0.998521	0.997665	0.999377	Train
SAGE	50	0.848415	0.820742	0.876088	Test
SAGE	50	0.888287	0.848405	0.928169	Held-out test
SAGE	100	1	1	1	Train
SAGE	100	0.850893	0.825609	0.876178	Test
SAGE	100	0.874354	0.830474	0.918234	Held-out test
SAGE	200	1	1	1	Train
SAGE	200	0.860456	0.834429	0.886483	Test
SAGE	200	0.885792	0.843956	0.927628	Held-out test
MIDAS GIN	50	0.989741	0.985927	0.993555	Train
MIDAS GIN	50	0.867478	0.836926	0.898031	Test
MIDAS GIN	50	0.908515	0.87062	0.94641	Held-out test
MIDAS GIN	200	0.983784	0.978718	0.988851	Train
MIDAS GIN	200	0.867559	0.837451	0.897667	Test
MIDAS GIN	200	0.893994	0.853299	0.934688	Held-out test
MIDAS GIN	100	0.926138	0.908163	0.944113	Train
MIDAS GIN	100	0.852362	0.820902	0.883822	Test
MIDAS GIN	100	0.862665	0.815236	0.910095	Held-out test
GIN MLP no proc concat	50	0.999909	0.999774	1	Train
GIN MLP no proc concat	50	0.84966	0.819543	0.879777	Test
GIN MLP no proc concat	50	0.874901	0.831368	0.918433	Held-out test
GIN MLP no proc concat	100	0.999645	0.999277	1	Train
GIN MLP no proc concat	100	0.833012	0.800108	0.865916	Test
GIN MLP no proc concat	100	0.873689	0.83218	0.915198	Held-out test
GIN MLP no proc concat	200	0.999779	0.999466	1	Train

GIN MLP no proc concat	200	0.818105	0.785814	0.850395	Test
GIN MLP no proc concat	200	0.857572	0.809429	0.905716	Held-out test
GIN MLP no proc	50	0.998453	0.997625	0.999281	Train
GIN MLP no proc	50	0.85907	0.82893	0.88921	Test
GIN MLP no proc	50	0.856601	0.799805	0.913397	Held-out test
GIN MLP no proc	100	0.997833	0.996713	0.998953	Train
GIN MLP no proc	100	0.863119	0.834544	0.891694	Test
GIN MLP no proc	100	0.88165	0.836116	0.927184	Held-out test
GIN MLP no proc	200	0.989258	0.984919	0.993597	Train
GIN MLP no proc	200	0.858282	0.826949	0.889614	Test
GIN MLP no proc	200	0.882341	0.838143	0.926538	Held-out test
GIN MLP	50	0.991885	0.9896	0.99417	Train
GIN MLP	50	0.869534	0.841703	0.897365	Test
GIN MLP	50	0.902548	0.86376	0.941336	Held-out test
GIN MLP	100	0.994361	0.992546	0.996177	Train
GIN MLP	100	0.86414	0.833565	0.894714	Test
GIN MLP	100	0.899996	0.861116	0.938877	Held-out test
GIN MLP	200	0.986858	0.982464	0.991252	Train
GIN MLP	200	0.870645	0.840763	0.900526	Test
GIN MLP	200	0.906199	0.869797	0.942601	Held-out test
GAT	50	0.920774	0.905745	0.935802	Train
GAT	50	0.850054	0.819038	0.88107	Test
GAT	50	0.883941	0.84129	0.926592	Held-out test
GAT	100	0.992992	0.991005	0.994979	Train
GAT	100	0.869834	0.841039	0.898629	Test
GAT	100	0.913045	0.879634	0.946457	Held-out test
GAT	200	0.968684	0.962249	0.97512	Train
GAT	200	0.85989	0.829819	0.889962	Test
GAT	200	0.904849	0.870929	0.938769	Held-out test

Supplementary Table 3: Top 15 predictions from MIDAS GIN

Gene	Predicted Score	Target Label
CCL5	0.999998	0
IL12RB1	0.999974	1
CCR1	0.999836	1
CD247	0.999758	0
CXCL10	0.999672	0
IL2RB	0.999562	1
IL23A	0.999361	0
IL7R	0.999344	0
IRF1	0.999252	0
CD86	0.999229	0
TLR4	0.999211	1

CTLA4	0.999133	1
IL12RB2	0.999012	0
IL12B	0.998975	0
PTPN6	0.998864	0

Supplementary Table 4: Performance of linear, tree-based, and MLP baselines.

Model	AUC	Lower CI	Upper CI	Set
MLP with graph loader	0.999881	0.999647	1	Train
MLP with graph loader	0.74151	0.702539	0.780481	Test
MLP with graph loader	0.803194	0.748004	0.858383	Held-out test
MLP	1	1	1	Train
MLP	0.746707	0.707742	0.785673	Test
MLP	0.794235	0.742115	0.846356	Held-out test
Elastic net	0.828247	0.796106	0.860388	Train
Elastic net	0.770266	0.731443	0.809089	Test
Elastic net	0.790861	0.731112	0.85061	Held-out test
XGBoost	0.931321	0.914813	0.94783	Train
XGBoost	0.779345	0.743683	0.815007	Test
XGBoost	0.796981	0.733569	0.860393	Held-out test

Supplementary Table 5: Optimal hyperparameters for linear, tree-based, and MLP baselines

Model	PyTorch/sklearn/XGBoost hyperparameter	Hyperparameter name	Value
MLP	Lr	Learning rate for the Adam optimiser	0.01
MLP	n_hidden	Number of neurons in hidden layers	256
MLP	dropout	Dropout probability	0.5
MLP	weight_decay	Weight decay (L2 penalty) for the Adam optimiser	0.000115
MLP	batch_size	Batch size	256

MLP	num_layers	Number of MLP layers	4
MLP	rate	Dropout rate for dropout layer	0.4
MLP with graph loader	lr	Learning rate for the Adam optimiser	0.02
MLP with graph loader	n_hidden	Number of neurons in hidden layers	16
MLP with graph loader	dropout	Dropout probability	0.2
MLP with graph loader	weight_decay	Weight decay (L2 penalty) for the Adam optimiser	0.000878
MLP with graph loader	batch_size	Batch size	64
MLP with graph loader	num_layers	Number of MLP layers	3
MLP with graph loader	rate	Dropout rate for dropout layer	0.1
XGBoost	booster	Which booster to use	gbtree
XGBoost	max_depth	Maximum tree depth for base learners	26
XGBoost	n_estimators	Number of gradient boosted trees	100
XGBoost	min_child_weight	Minimum sum of instance weight (Hessian) needed in a child.	10
XGBoost	scale_pos_weight	Controls the balance of positive and negative weights	3
XGBoost	subsample	Subsample ratio of the training instances.	0.635382
XGBoost	colsample_bytree	Subsample ratio of columns when constructing each tree	0.681178
XGBoost	colsample_bylevel	Subsample ratio of columns for each level	0.60972

XGBoost	colsample_bynode	Subsample ratio of columns for each node (split)	0.283404
XGBoost	eta	Boosting learning rate	5.45E-08
XGBoost	gamma	Minimum loss reduction required to make a further partition on a leaf node of the tree	2.90E-05
XGBoost	grow_policy	Controls the way new nodes are added to the tree.	depthwise
XGBoost	reg_alpha	L1 regularization term on weights	5.92E-07
XGBoost	reg_lambda	L2 regularization term on weights	1.976838
elastic net	C	Inverse of regularisation strength	0.002936
elastic net	l1_ratio	Mixing parameter defining contribution of L1 and L2 to the penalty	0.03

Supplementary Table 6: Pathways enriched within MIDAS GIN predictions

Gene-set enrichment analysis (GSEA). Attached as a separate Excel file.

Supplementary Table 7: Permutation feature importance across specificity thresholds

Attached as a separate Excel file.

Supplementary Table 8: Shortlisted candidate targets

Gene	Predicted Score	Target Label
ELP2	0.984909	0
TICAM1	0.98269	0
GNA14	0.981949	0
TPST2	0.981656	0
SHC1	0.980343	0
PDGFRB	0.980037	0
SOCS3	0.979776	0
PGF	0.978615	0
TRAF5	0.971975	0
TXK	0.971747	0
IRS2	0.971143	0
LEPR	0.970358	0
IDE	0.969673	0
DPP8	0.96841	0
INPP5D	0.968262	0
OSM	0.966355	0
IGFBP7	0.966159	0
OSMR	0.965082	0
CRKL	0.962411	0
SLAMF6	0.958312	0
MAVS	0.958135	0
NLRP10	0.956361	0
SUSD2	0.952084	0
TGFBR3	0.945654	0
LEPROTL1	0.945122	0
LCN2	0.942245	0
PLAUR	0.941637	0
STAP2	0.941099	0
ANGPT4	0.939125	0
PTK2B	0.938305	0
SH2D2A	0.936432	0

CMKLR2	0.935811	0
PXN	0.935328	0
SDC4	0.93406	0
BID	0.933402	0
FMOD	0.931877	0
HAX1	0.927557	0
CEBPB	0.921995	0
INHBE	0.917107	0
CREB3	0.91236	0
DOK1	0.909834	0
PTPN1	0.908543	0
PML	0.908153	0

Supplementary Table 9: Summary patient clinical characteristics

Patient Characteristics	Total N=8
Median age (range)	69 (39-83)
Male sex [n (%)]	5 (63)
Melanoma subtype [n (%)]	
- Cutaneous	4 (50)
- Melanoma of Unknown Primary	1 (13)
- Acral	2 (25)
- Uveal	1 (13)
Anatomical Site of Melanoma Sampled [n (%)]	
- Lymph node	5 (63)
- Local recurrence	1 (13)
- Axilla	1 (13)
- Liver	1 (13)
Disease Stage at Sampling [n (%)]	
- Stage 3	6 (75)
- Stage 4	2 (25)
Prior systemic therapy [n (%)]	
- Nil	6 (75)
- Anti-PD-1 (adjuvant)	2 (25)
Subsequent systemic therapy [n (%)]	
- Anti-PD1 (adjuvant)	3 (38)
- Ipilimumab Nivolumab	2 (25)
- Tebentafusp	1 (13)
- Nil known	2 (25)

Percentages in brackets are rounded to two significant figures.

Supplementary Table 10: Individual patient clinical characteristics

Attached as a separate Excel file.

Supplementary Table 11: MIDAS GIN optimal hyperparameters

GIN PyTorch hyperparameters	GIN hyperparameter name	Optimal value
lr	Learning rate for the Adam optimiser	0.01
n_hidden	Number of neurons in hidden layers	16
dropout	Dropout probability	0.5
weight_decay	Weight decay (L2 penalty) for the Adam optimiser	5.21×10^{-4}
batch_size	Batch size	256
num_layers	Number of GNN message passing layers	2
eps	Epsilon in eq1 and eq2 in the main text	0.15
train_eps	Whether to train the epsilon parameter in eq1 and eq2 in the main text	False
num_layers_pre	Number of pre-processing layers	1
num_layers_post	Number of post-processing layers	1
rate	Dropout rate for dropout layer	0.2

Supplementary Figures

Supplementary Figure 1: Performance of different GNN models at identifying known immuno-oncology targets in clinical development

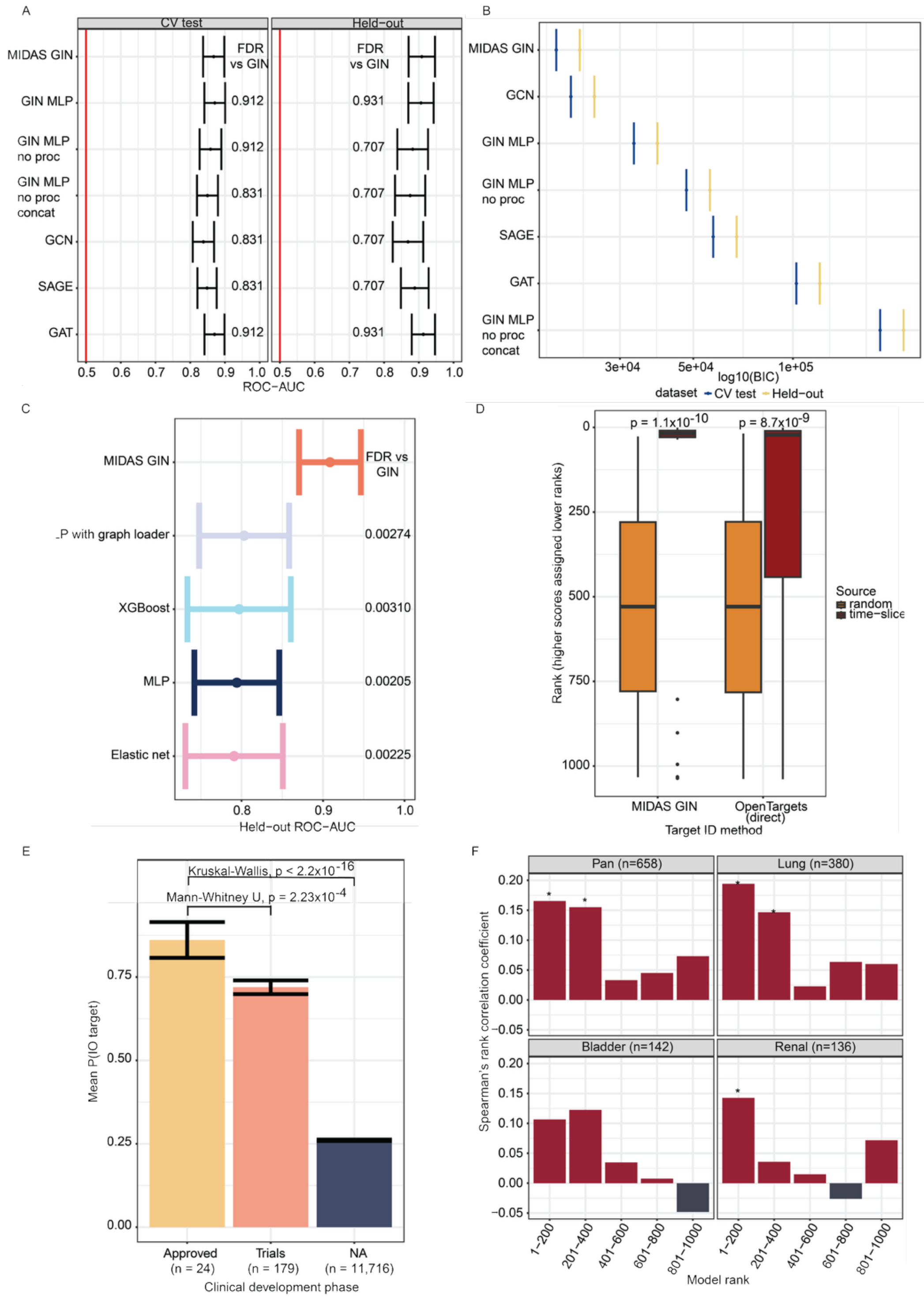

A) Numbers indicate the FDR-corrected Delong's test p-value comparing model performance to the GIN. Red line indicates random performance. CV test corresponds to predictions for n = 8,933 genes, whilst held-out corresponds to predictions for the n = 2,986 held-out genes. CV

test = Cross-validation test set; GAT = graph attention network; GCN = graph convolutional network; GIN = graph isomorphism network; GIN MLP = GIN using multi-layer perceptrons; GIN MLP no proc = GIN without using any pre- or post-processing layers; GIN MLP no proc concat = GIN without using any pre- or post-processing layers and implementing global concatenation; GNN = graph neural network; ROC-AUC = Area under the receiver-operator characteristic; SAGE = graph sample and aggregate network.

B) Model selection using the Bayesian Information Criterion. Data are represented as the mean BIC $\pm$ standard deviation across the n = 100 CV fold models developed for each GNN variant. CV test = Cross-validation test set. GNN = graph neural network.

C) Benchmarking MIDAS GIN against alternative approaches that do not use any message passing dynamics. Each alternative algorithm was trained using the same cross-validation and hyperparameter optimisation routines as for the MIDAS GIN. All models were trained using the same feature matrix as the GNN node feature matrix. Since the alternate models do not incorporate GiG network topology, the GiG node degree was concatenated to the node feature matrix. FDR for comparison against MIDAS GIN are shown (Delong's test) using predictions computed on the held-out test set (n = 2,986 genes). MLP = multi-layer perceptron; MLP with graph loader = MLP where the MIDAS GIN graph neighbour loader was used to inform selection of batches for training.

D) Distribution of rankings of time-sliced targets compared to random genes between MIDAS GIN and OpenTargets (direct evidence). Output scores from both methods were ranked such that higher scores were assigned lower ranks. Time-sliced targets numbered n = 36 for MIDAS GIN and n = 39 for OpenTargets (direct), with the difference stemming from certain genes being represented in only one of the two tested methods. P-values are derived from two-sided Mann-Whitney U tests. The centre line indicates the median, box borders represent the upper and lower quartiles, whiskers represent the inter-quartile range multiplied by 1.5, and black points are outliers

E) MIDAS model discriminates genes that are approved for use in clinical practice. Time-sliced targets (genes that go on to be addressed in clinical trials in time-sliced data) are included within the "Trials" category. NA indicates genes that did not have phase information available. Data are presented as mean values $\pm$ standard error. Exact p-value for the Kruskal-Walis test: $2.54x10^{-79}$. The comparison between approved targets and those undergoing clinical trials was assessed using a two-sided Mann-Whitney U test.

F) Correlation between model predictions and Wald test statistic for DEGs. Stars indicate significant correlations (p<0.05, Spearman's rank). Exact p-values for pan: 0.0194 for ranks 1-

200 and 0.0291 for ranks 201-400; lung: p = 5.89×10^{-3} for ranks 1-200 and 0.0394 for ranks 201-400; renal: p = 0.0441 for ranks 1-200. Sample numbers for each cancer type are annotated.

Supplementary Figure 2: Meta-learning framework for immuno-oncology target discovery

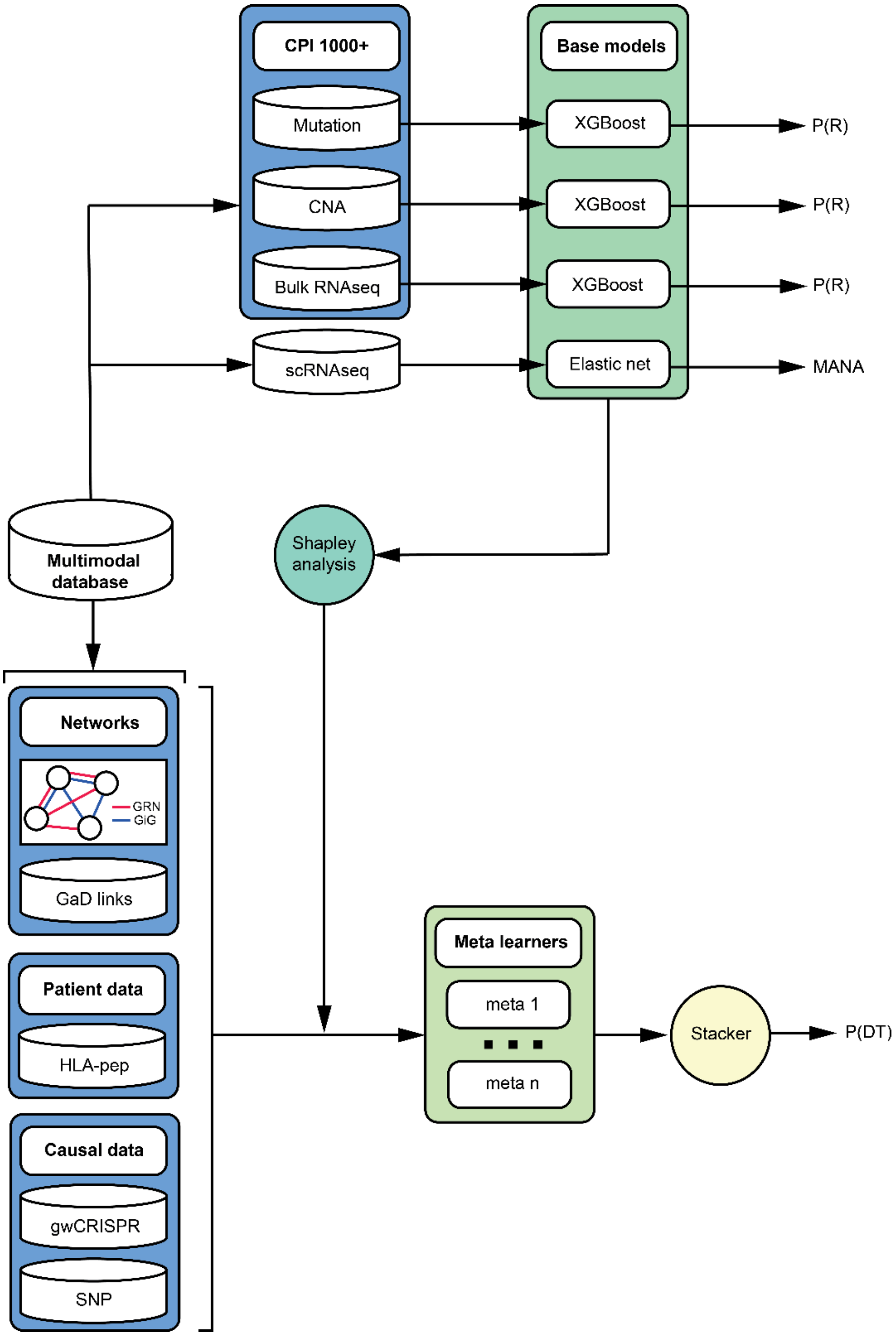

n = number of meta-learners. gwCRISPR = genome-wide CRISPR co-cultures, DT = drug target, GaD = gene-disease associations, GiG = gene-interaction-gene network, GRN = gene regulatory network,

MANA = mutation-associated neoantigen score, R = response to immune checkpoint inhibitor therapy, SNP = single nucleotide polymorphism.

Supplementary Figure 3: Node feature permutation importance across train-validation splits

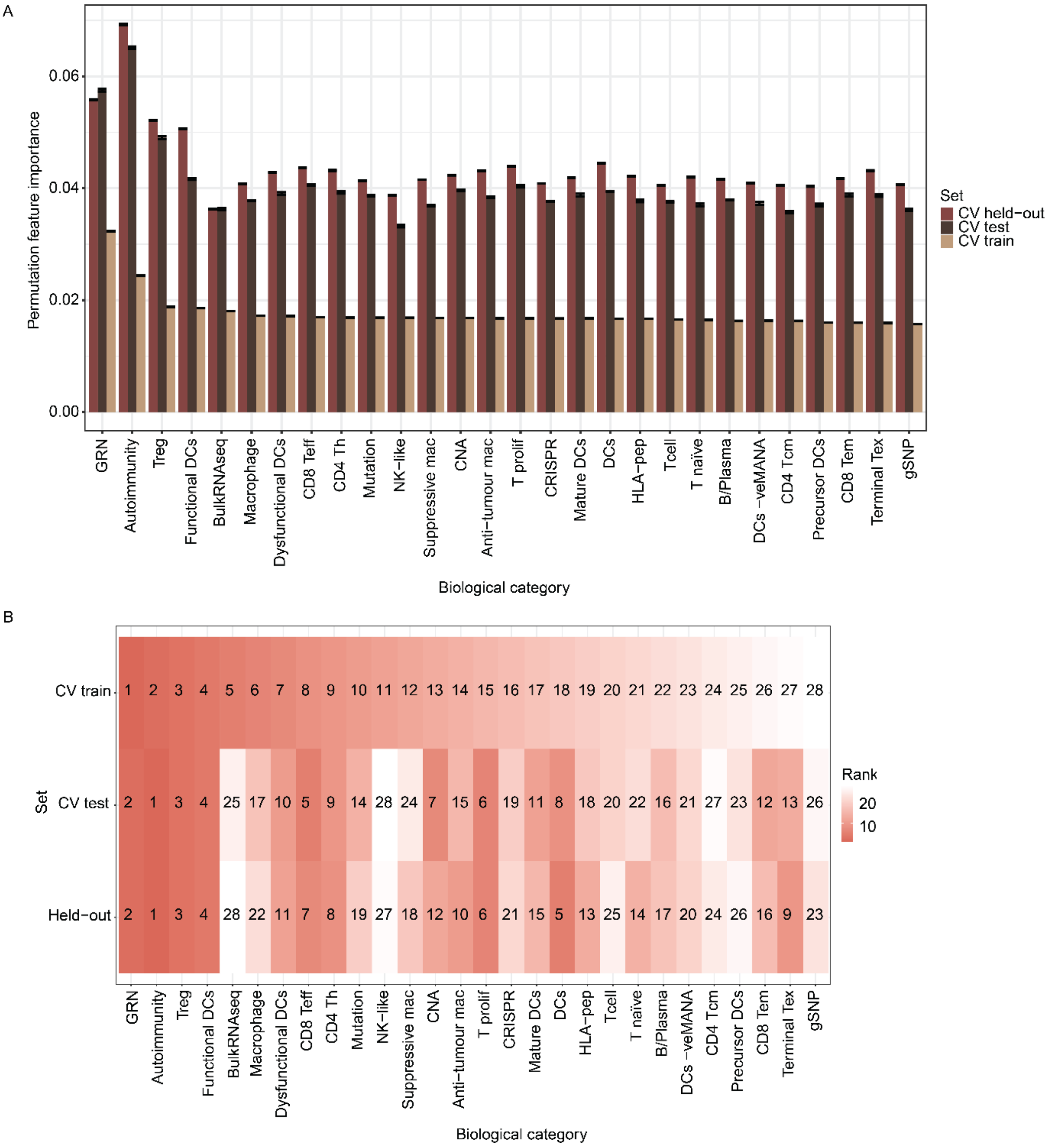

A) Permutation feature importance by biological category across CV train, CV test, and held-out sets. Feature importance is defined as the absolute difference between the ROC-AUC on the original data and the permuted version, for each of CV train, CV test, and held-out sets, respectively. Data are presented as the mean ± standard error of the permutation feature importance across 500 permutations and 100 CV fold models and features that map to each

biological category. For visual clarity, biological categories are ordered by their ranking in the CV train set.

B) Ranked permutation importance of input features, grouped by biological category. Feature importance is defined as the absolute difference between the ROC-AUC on the original data and the permuted version, for each of CV train, CV test, and held-out sets, respectively. For visual clarity, biological categories are ordered by their ranking in the CV train set.

Supplementary Figure 4: Pathway permutation approach

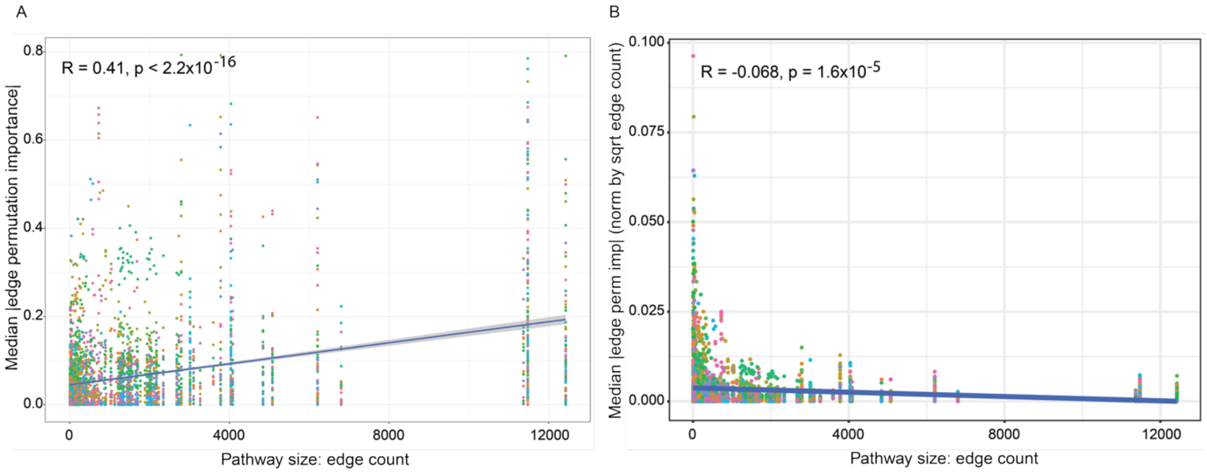

A) Pathway permutation importance depends strongly on pathway size. Each point represents a pathway-target pair for the set of known immunotherapy targets. The Spearman rank correlation coefficient and p-value (exact $p = 6.35 \times 10^{-159}$) are shown.

B) Normalising for pathway size effectively corrects for the pathway permutation importance. Each point represents a pathway-target pair. The Spearman rank correlation coefficient and p-value are shown.

Supplementary figure 5: OSM expression is associated with altered T cell scores by CIBERSORT analysis of bulk transcriptomics data

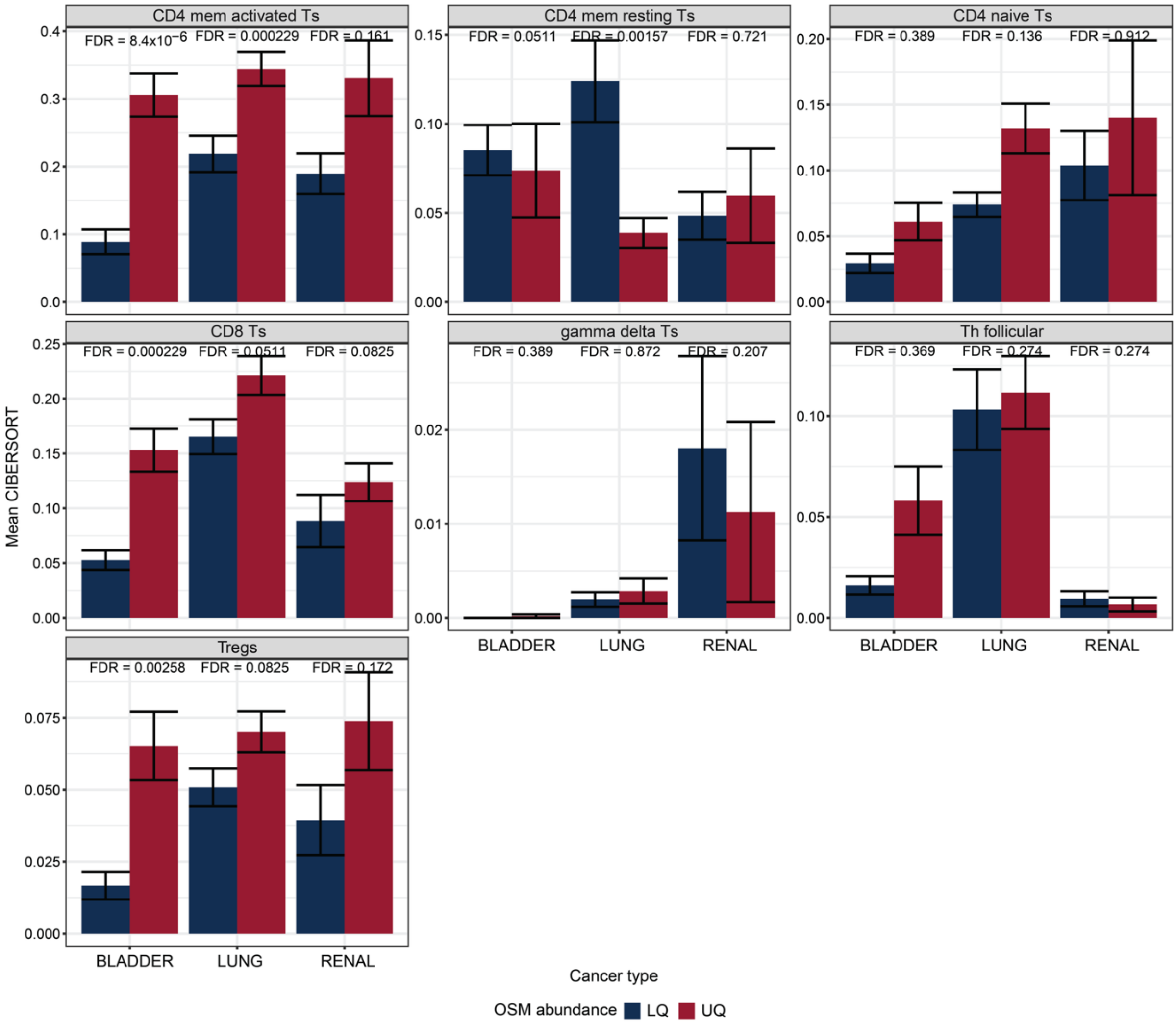

Mean CIBERSORT deconvolution scores for T cell subsets by OSM expression. OSM expression is discretised into upper quartile (UQ) and lower quartile (LQ) and assessed using a two-sided Mann Whitney test. P-values were adjusted for multiple testing. Only patients whose expression profiles were successfully deconvolved ($p < 0.05$) are included. Error bars represent the mean ± standard error. Expression is quantified using transcripts per million (TPM). Sample numbers: bladder = 142, lung = 380, renal = 136.

Supplementary figure 6: OSMR expression is associated with altered T cell scores by CIBERSORT analysis of bulk transcriptomics data

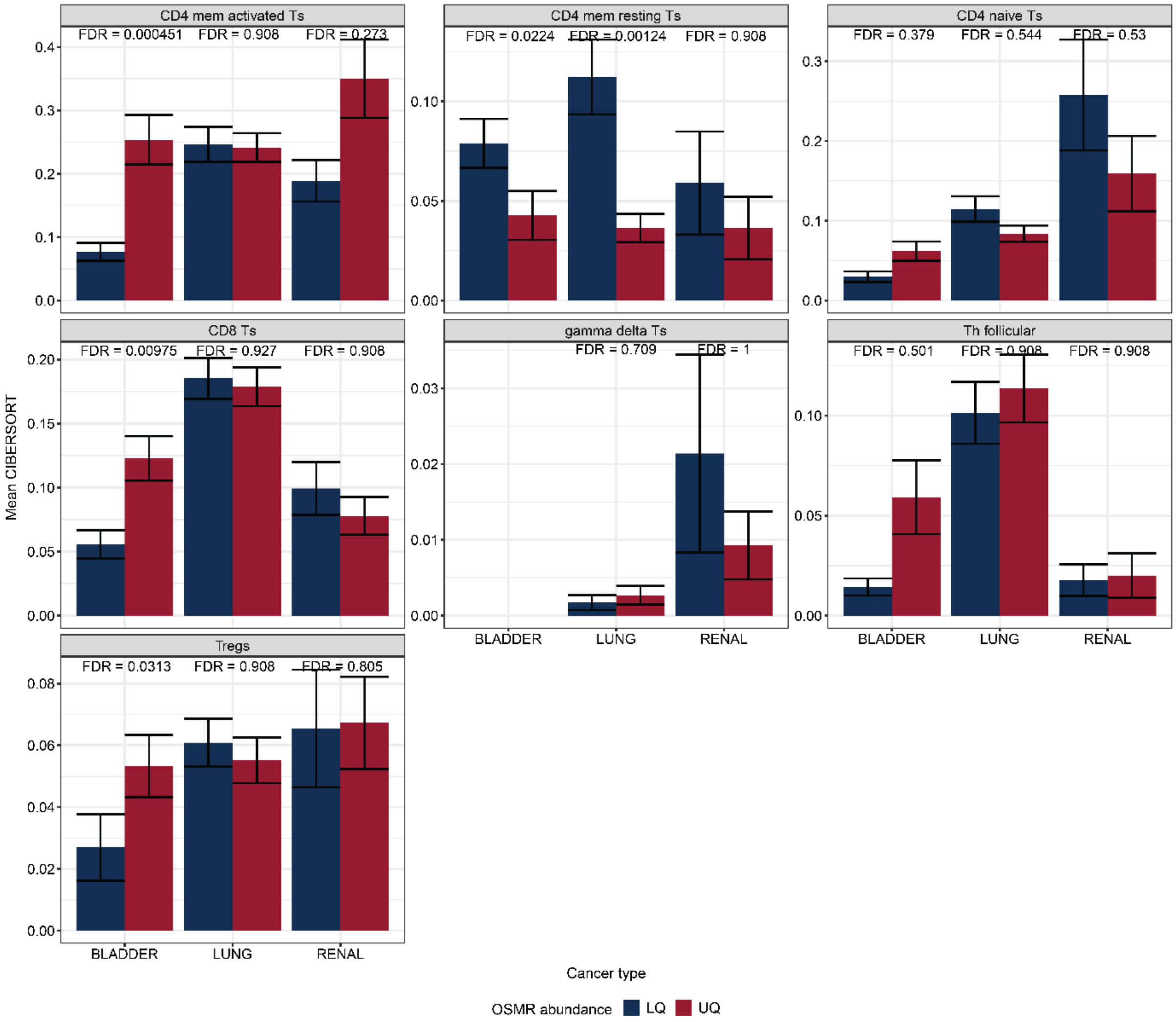

Mean CIBERSORT deconvolution scores for T cell subsets by OSMR expression. OSMR expression is discretised into upper quartile (UQ) and lower quartile (LQ) and assessed using a two-sided Mann Whitney test. P-values were adjusted for multiple testing. Only patients whose expression profiles were successfully deconvolved ($p < 0.05$) are included. Error bars represent the mean $\pm$ standard error. Expression is quantified using transcripts per million (TPM). Sample numbers: bladder = 142, lung = 380, renal = 136.

Supplementary figure 7: OSMR expression is associated with altered macrophage cell scores by CIBERSORT analysis of bulk transcriptomics data

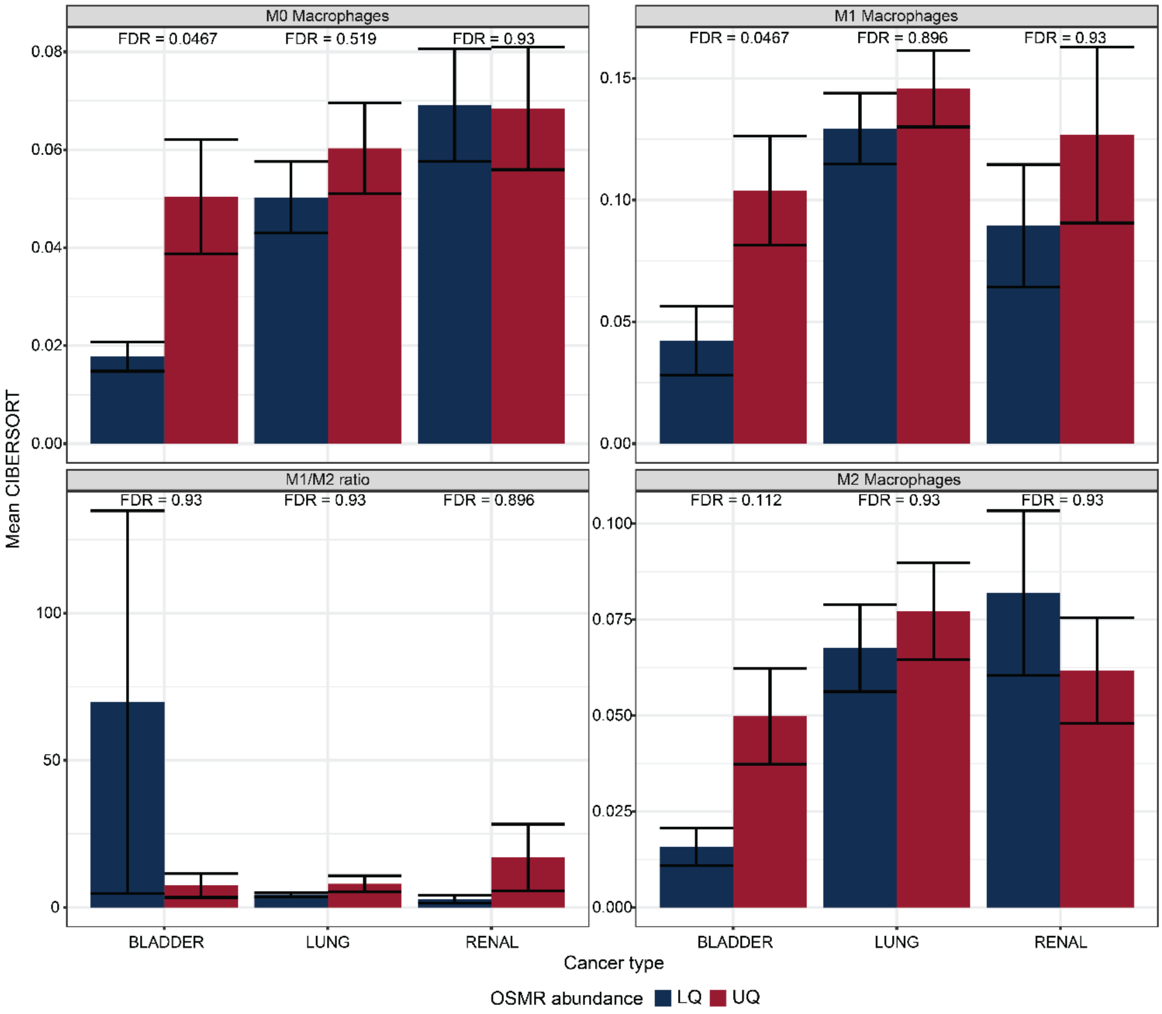

Mean CIBERSORT deconvolution scores for macrophage subsets by OSMR expression. OSMR expression is discretised into upper quartile (UQ) and lower quartile (LQ) and assessed using a two-sided Mann Whitney test. P-values were adjusted for multiple testing. Only patients whose expression profiles were successfully deconvolved ($p < 0.05$) are included. Error bars represent the mean $\pm$ standard error. Expression is quantified using transcripts per million (TPM). Sample numbers: bladder = 142, lung = 380, renal = 136.

Supplementary figure 8: OSM expression is associated with altered macrophage cell scores by CIBERSORT analysis of bulk transcriptomics data

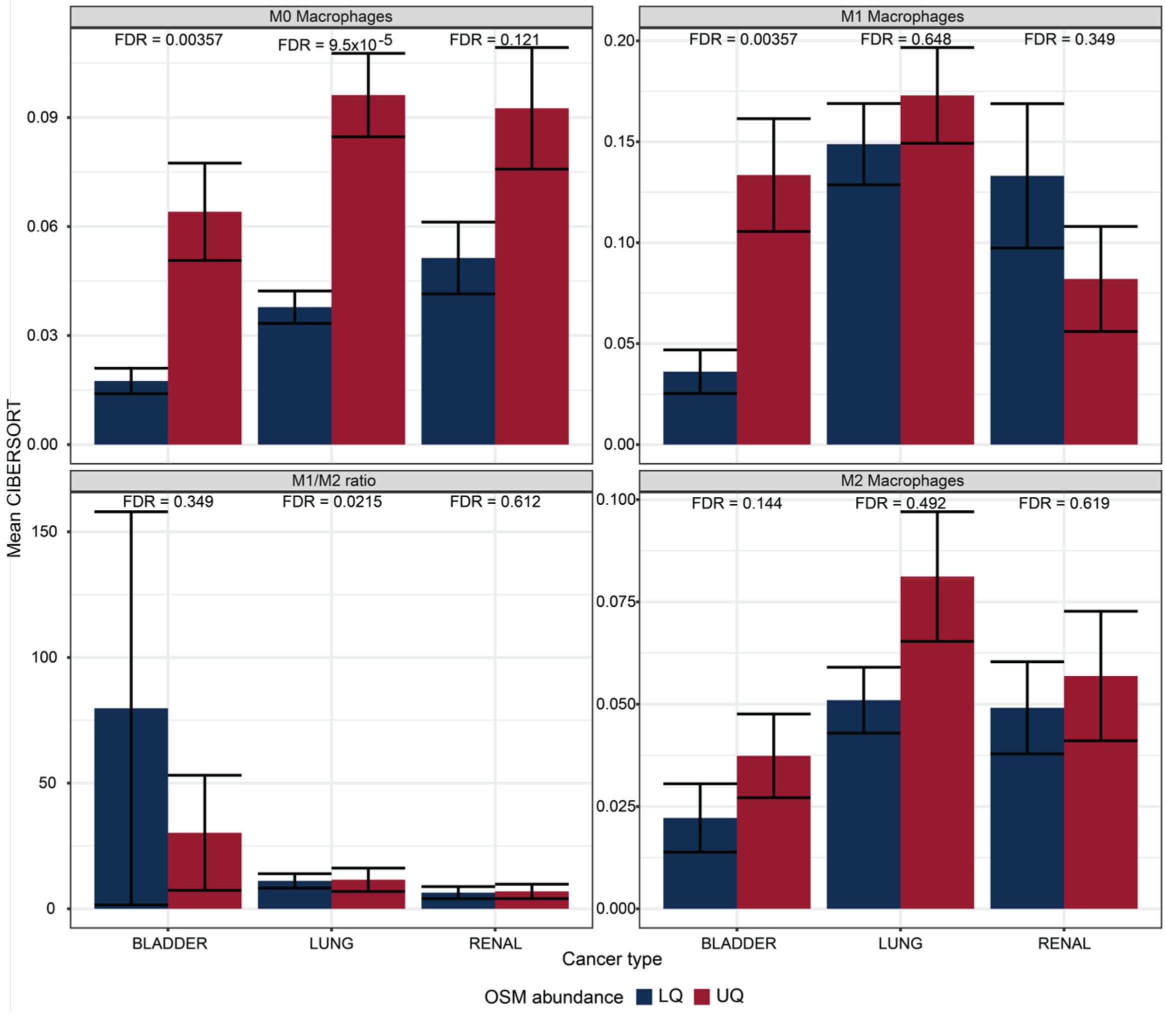

Mean CIBERSORT deconvolution scores for macrophage subsets by OSM expression. OSM expression is discretised into upper quartile (UQ) and lower quartile (LQ) and assessed using a two-sided Mann Whitney test. P-values were adjusted for multiple testing. Only patients whose expression profiles were successfully deconvolved ($p < 0.05$) are included. Error bars represent the mean ± standard error. Expression is quantified using transcripts per million (TPM). Sample numbers: bladder = 142, lung = 380, renal = 136.

Supplementary Figure 9: Validation of genes with high topological specificity scores in scRNA-seq data

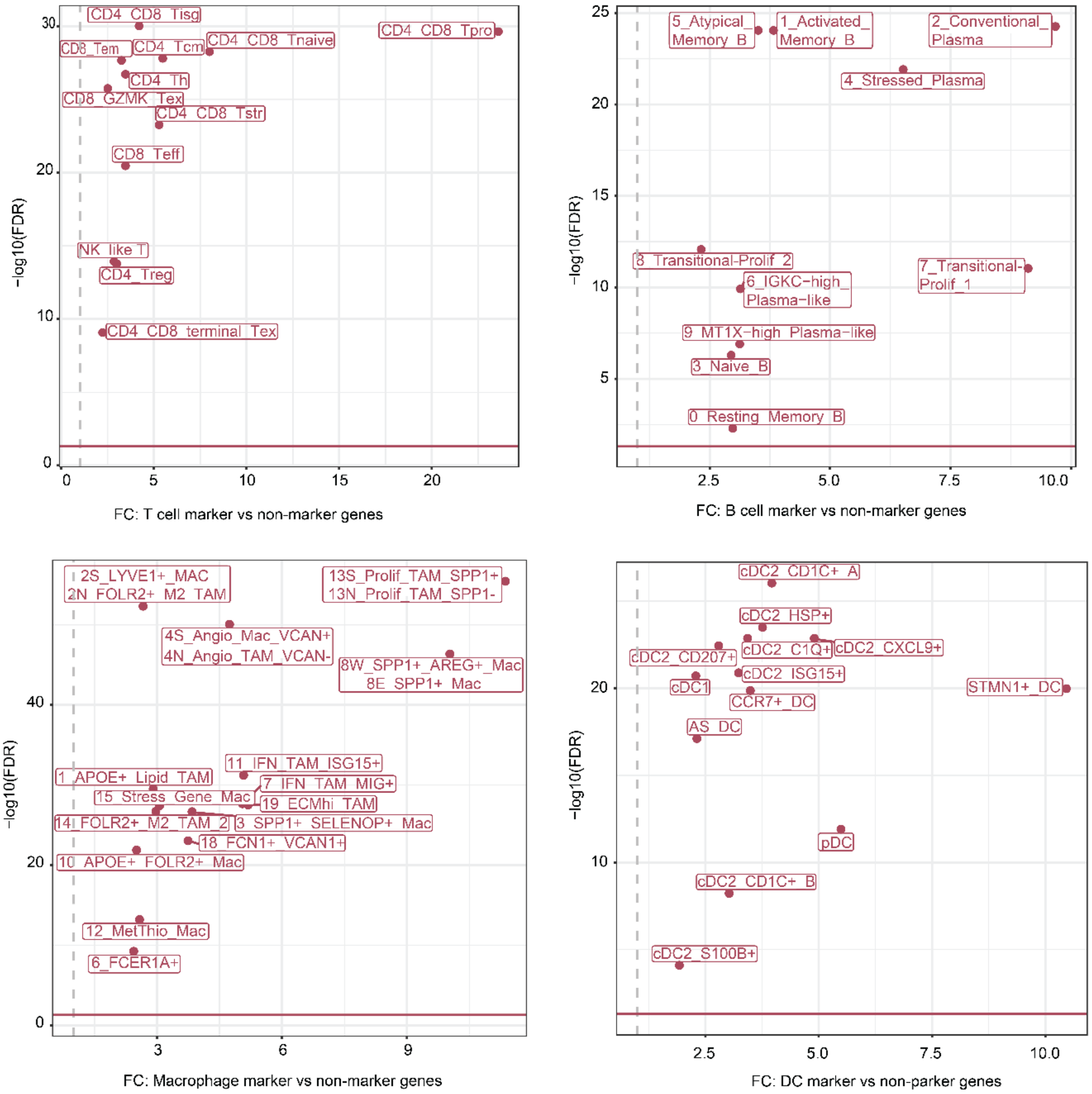

Fold change of topological specificity scores, for each cell subtype, for marker genes (which delineate that cell subtypes) compared to non-marker genes. The red line indicates the significance threshold ($-\log_{10}(\text{FDR} = 0.05)$) for the two-tailed Mann-Whitney U test comparing the scores in marker vs non-marker genes.

Supplementary Figure 10: Comparison of overrepresented pathways amongst the top 300 ranked genes and the shortlisted candidates

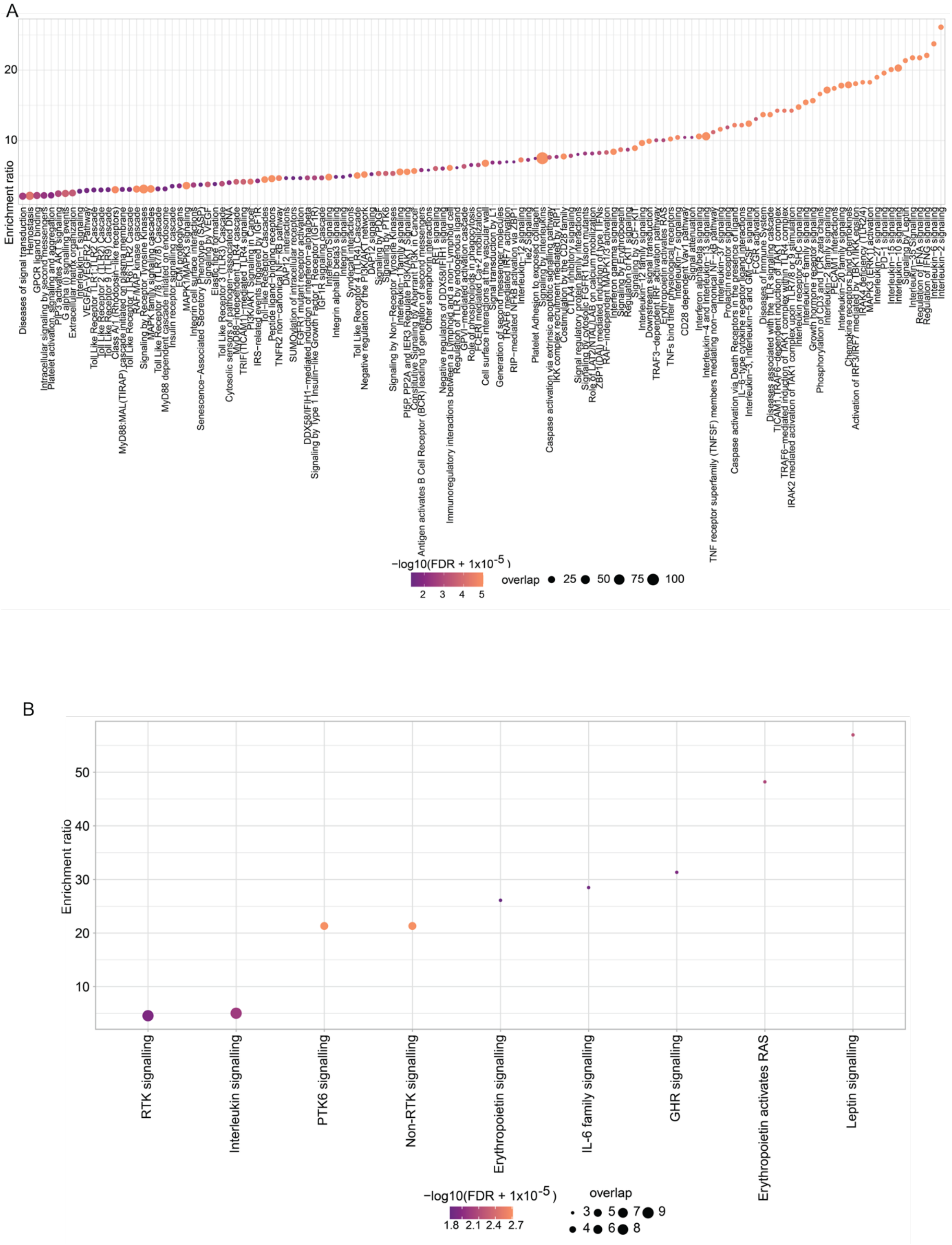

A) Significantly overrepresented pathways in top 300 ranked predictions. Significance was determined using an FDR threshold of 0.05.

B) Significantly overrepresented pathways in the shortlisted genes following exclusions. Significance was determined using an FDR threshold of 0.05.

Supplementary Figure 11: Representative gating strategy for PDE assays

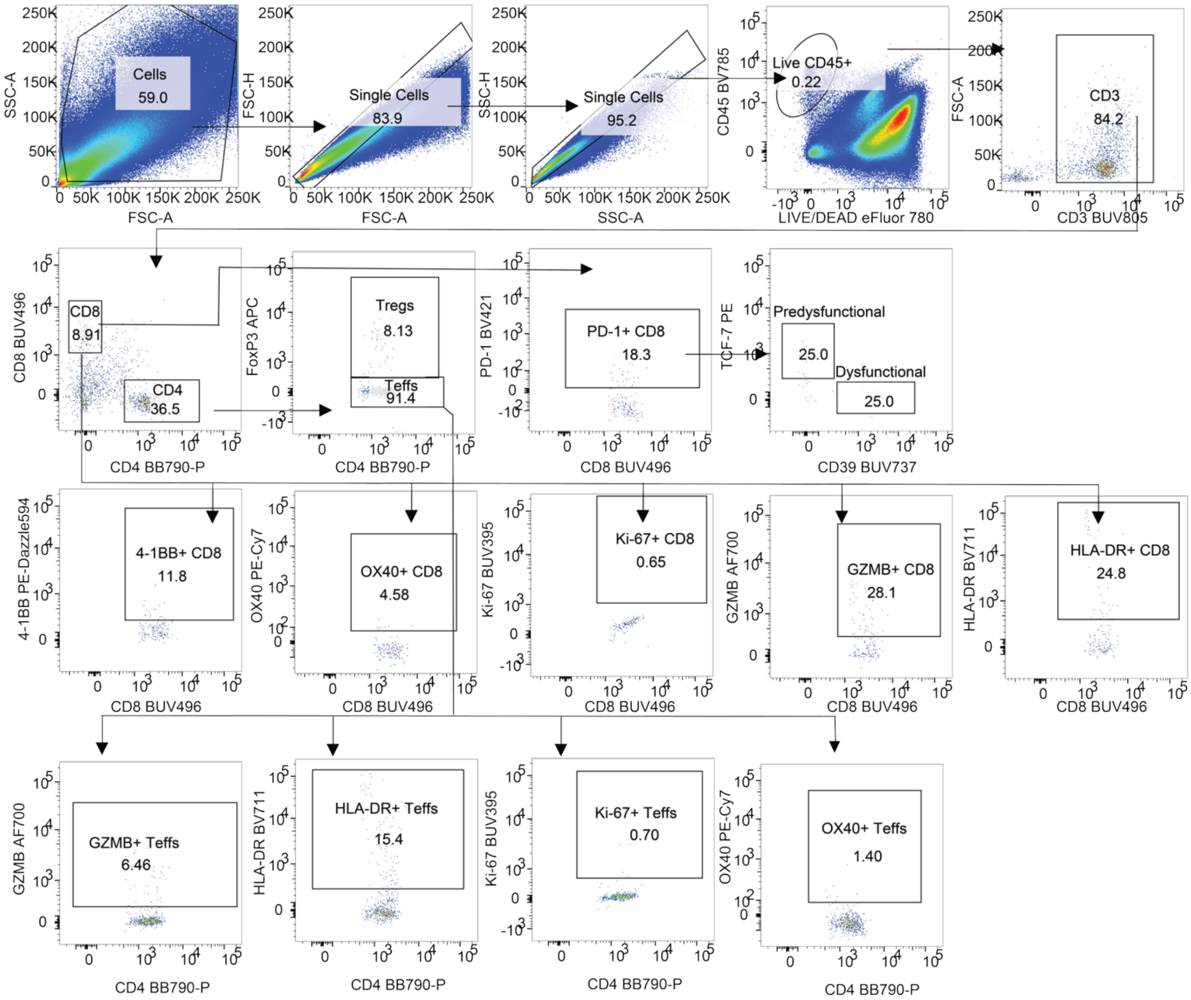

The immune population within PDEs was gated as live, CD45+ single cells. T cells (CD3+) were divided into CD4+ and CD8+ populations, then analysed for expression of markers of activation (PD-1, HLA-DR, OX40, 4-1BB), proliferation (Ki-67), and cytotoxicity (GZMB). In addition, CD4+ T cells were identified as regulatory and non-regulatory (Tregs and Teffs) based on expression of FoxP3, while CD8+ T cells were identified as pre-dysfunctional (PD-1+ TCF-7+ CD39-) and dysfunctional (PD-1+ TCF-7- CD39+). Teff = effector T cell, GZMB = granzyme B, Treg = regulatory T cell, FSC = forward scatter, SSC = side scatter.

References

Akiba, T., Sano, S., Yanase, T., Ohta, T. & Koyama, M. Optuna: A next-generation hyperparameter optimization framework. In Proceedings of the 25th ACM SIGKDD International Conference on Knowledge Discovery & Data Mining (eds Teredesai, A., Kumar, V., Li, Y., Rosales, R., Terzi, E. & Karypis, G.) 2623-2631 (ACM, 2019)

Bronstein, M. M., Bruna, J., Cohen, T., & Veličković, P. (2021). *Geometric Deep Learning: Grids, Groups, Graphs, Geodesics, and Gauges*. https://arxiv.org/abs/2104.13478v2

Bulik-Sullivan, B., Busby, J., Palmer, C. D., Davis, M. J., Murphy, T., Clark, A., Busby, M., Duke, F., Yang, A., Young, L., Ojo, N. C., Caldwell, K., Abhyankar, J., Boucher, T., Hart, M. G., Makarov, V., De Montpreville, V. T., Mercier, O., Chan, T. A., ... Yelensky, R. (2018). Deep learning using tumor HLA peptide mass spectrometry datasets improves neoantigen identification. *Nature Biotechnology 2018 37:1*, *37*(1), 55–63. https://doi.org/10.1038/nbt.4313

Caushi, J. X., Zhang, J., Ji, Z., Vaghasia, A., Zhang, B., Hsiue, E. H. C., Mog, B. J., Hou, W., Justesen, S., Blosser, R., Tam, A., Anagnostou, V., Cottrell, T. R., Guo, H., Chan, H. Y., Singh, D., Thapa, S., Dykema, A. G., Burman, P., ... Smith, K. N. (2021). Transcriptional programs of neoantigen-specific TIL in anti-PD-1-treated lung cancers. *Nature 2021 596:7870*, *596*(7870), 126–132. https://doi.org/10.1038/s41586-021-03752-4

Chen, T., & Guestrin, C. (2016). XGBoost: A Scalable Tree Boosting System. *Proceedings of the ACM SIGKDD International Conference on Knowledge Discovery and Data Mining*, *13-17-August-2016*, 785–794. https://doi.org/10.1145/2939672.2939785

Fehlings, M., Jhunjhunwala, S., Kowanetz, M., O'Gorman, W. E., Hegde, P. S., Sumatoh, H., Lee, B. H., Nardin, A., Becht, E., Flynn, S., Ballinger, M., Newell, E. W., & Yadav, M. (2019). Late-differentiated effector neoantigen-specific CD8+ T cells are enriched in peripheral blood of non-small cell lung carcinoma patients responding to atezolizumab treatment. *Journal for ImmunoTherapy of Cancer*, *7*(1), 249. https://doi.org/10.1186/S40425-019-0695-9

Fehlings, M., Simoni, Y., Penny, H. L., Becht, E., Loh, C. Y., Gubin, M. M., Ward, J. P., Wong, S. C., Schreiber, R. D., & Newell, E. W. (2017). Checkpoint blockade immunotherapy reshapes the high-dimensional phenotypic heterogeneity of murine intratumoural neoantigen-specific CD8+ T cells. *Nature Communications 2017 8:1*, *8*(1), 1–12. https://doi.org/10.1038/s41467-017-00627-z

George, S., Miao, D., Demetri, G. D., Adeegbe, D., Rodig, S. J., Shukla, S., Lipschitz, M., Amin-Mansour, A., Raut, C. P., Carter, S. L., Hammerman, P., Freeman, G. J., Wu, C. J., Ott, P. A., Wong, K. K., & Van Allen, E. M. (2017). Loss of PTEN Is Associated with Resistance to Anti-PD-1 Checkpoint Blockade Therapy in Metastatic Uterine Leiomyosarcoma. *Immunity*, *46*(2), 197–204. https://doi.org/10.1016/J.IMMUNI.2017.02.001

Hamilton, W. L., Ying, R., & Leskovec, J. Inductive Representation Learning on Large Graphs. In Proceedings of the 31st International Conference on Neural Information Processing Systems 1025–1035 (Curran Associates Inc., 2017).

Hao, Y., Hao, S., Andersen-Nissen, E., Mauck, W. M., Zheng, S., Butler, A., Lee, M. J., Wilk, A. J., Darby, C., Zager, M., Hoffman, P., Stoeckius, M., Papalexi, E., Mimitou, E. P., Jain, J., Srivastava, A., Stuart, T., Fleming, L. M., Yeung, B., ... Satija, R. (2021). Integrated analysis of multimodal single-cell data. *Cell*, *184*(13), 3573-3587.e29.

https://doi.org/10.1016/J.CELL.2021.04.048/ATTACHMENT/1E5EB5C1-59EE-4B2B-8BFA-14B48A54FF8F/MMC3.XLSX

Himmelstein, D. S., & Baranzini, S. E. (2015). Heterogeneous Network Edge Prediction: A Data Integration Approach to Prioritize Disease-Associated Genes. *PLOS Computational Biology*, *11*(7), e1004259. https://doi.org/10.1371/JOURNAL.PCBI.1004259

Kamphorst, A. O., Pillai, R. N., Yang, S., Nasti, T. H., Akondy, R. S., Wieland, A., Sica, G. L., Yu, K., Koenig, L., Patel, N. T., Behera, M., Wu, H., McCausland, M., Chen, Z., Zhang, C., Khuri, F. R., Owonikoko, T. K., Ahmed, R., & Ramalingam, S. S. (2017). Proliferation of PD-1+ CD8 T cells in peripheral blood after PD-1-targeted therapy in lung cancer patients. *Proceedings of the National Academy of Sciences of the United States of America*, *114*(19), 4993–4998. https://doi.org/10.1073/PNAS.1705327114/SUPPL_FILE/PNAS.201705327SI.PDF

Lemaitre, G., Nogueira, F., & Aridas char, C. K. (2017). Imbalanced-learn. *The Journal of Machine Learning Research*, *18*, 1–5. https://doi.org/10.5555/3122009.3122026

Litchfield, K., Reading, J. L., Puttick, C., Thakkar, K., Abbosh, C., Bentham, R., Watkins, T. B. K., Rosenthal, R., Biswas, D., Rowan, A., Lim, E., Al Bakir, M., Turati, V., Guerra-Assunção, J. A., Conde, L., Furness, A. J. S., Saini, S. K., Hadrup, S. R., Herrero, J., … Swanton, C. (2021). Meta-analysis of tumor- and T cell-intrinsic mechanisms of sensitization to checkpoint inhibition. *Cell*, *184*(3), 596-614.e14. https://doi.org/10.1016/J.CELL.2021.01.002

Love, M. I., Huber, W., & Anders, S. (2014). Moderated estimation of fold change and dispersion for RNA-seq data with DESeq2. *Genome Biology*, *15*(12), 1–21. https://doi.org/10.1186/S13059-014-0550-8/FIGURES/9

Lundberg, S. M. & Lee, S.-I. A unified approach to interpreting model predictions. In Advances in Neural Information Processing Systems 30 (eds Guyon, I., von Luxburg, U., Bengio, S., Wallach, H., Fergus, R., Vishwanathan, S. V. N. & Garnett, R.) 4765-4774 (Curran Associates, Inc., 2017)

Mayr, C., & Bartel, D. P. (2009). Widespread Shortening of 3'UTRs by Alternative Cleavage and Polyadenylation Activates Oncogenes in Cancer Cells. *Cell*, *138*(4), 673–684. https://doi.org/10.1016/J.CELL.2009.06.016/ATTACHMENT/71585A25-AD01-459B-9BC5-15932BD6EC20/MMC1.PDF

Mehmood, A., Ali, M. S., Li, D., Kaushik, A. C., & Wei, D. Q. (2024). Unveiling the Therapeutic Potential of Paclitaxel Combinations Against Breast Carcinoma and Identification of In Vivo Biomarkers. *Chemical Biology & Drug Design*, *104*(3), e14627. https://doi.org/10.1111/CBDD.14627

Oliveira, G., Stromhaug, K., Klaeger, S., Kula, T., Frederick, D. T., Le, P. M., Forman, J., Huang, T., Li, S., Zhang, W., Xu, Q., Cieri, N., Clauser, K. R., Shukla, S. A., Neuberg, D., Justesen, S., MacBeath, G., Carr, S. A., Fritsch, E. F., … Wu, C. J. (2021). Phenotype, specificity and avidity of antitumour CD8+ T cells in melanoma. *Nature 2021 596:7870*, *596*(7870), 119–125. https://doi.org/10.1038/s41586-021-03704-y

Pedregosa, F., Michel, V., Grisel, O., Blondel, M., Prettenhofer, P., Weiss, R., Vanderplas, J., Cournapeau, D., Pedregosa, F., Varoquaux, G., Gramfort, A., Thirion, B., Grisel, O., Dubourg, V., Passos, A., Brucher, M., Perrot, M., & Duchesnay, É. (2011). Scikit-learn: Machine Learning in Python. *Journal of Machine Learning Research*, *12*(85), 2825–2830. http://jmlr.org/papers/v12/pedregosa11a.html

Pittet, M. J., Michielin, O., & Migliorini, D. (2022). Clinical relevance of tumour-associated macrophages. *Nature Reviews Clinical Oncology 2022 19:6*, *19*(6), 402–421. https://doi.org/10.1038/s41571-022-00620-6

Rizvi, N. A., Hellmann, M. D., Snyder, A., Kvistborg, P., Makarov, V., Havel, J. J., Lee, W., Yuan, J., Wong, P., Ho, T. S., Miller, M. L., Rekhtman, N., Moreira, A. L., Ibrahim, F., Bruggeman, C.,

Gasmi, B., Zappasodi, R., Maeda, Y., Sander, C., … Chan, T. A. (2015). Mutational landscape determines sensitivity to PD-1 blockade in non-small cell lung cancer. *Science*, *348*(6230), 124–128. https://doi.org/10.1126/SCIENCE.AAA1348/SUPPL_FILE/RIZVI-SM.PDF

Schulte-Sasse, R., Budach, S., Hnisz, D., & Marsico, A. (2021). Integration of multiomics data with graph convolutional networks to identify new cancer genes and their associated molecular mechanisms. *Nature Machine Intelligence 3*, 513–526. https://doi.org/10.1038/s42256-021-00325-y

Shwartz-Ziv, R., & Armon, A. (2022). Tabular data: Deep learning is not all you need. *Information Fusion*, *81*, 84–90. https://doi.org/10.1016/J.INFFUS.2021.11.011

Van Rooij, N., Van Buuren, M. M., Philips, D., Velds, A., Toebes, M., Heemskerk, B., Van Dijk, L. J. A., Behjati, S., Hilkmann, H., El Atmioui, D., Nieuwland, M., Stratton, M. R., Kerkhoven, R. M., Keşmir, C., Haanen, J. B., Kvistborg, P., & Schumacher, T. N. (2013). Tumor exome analysis reveals neoantigen-specific T-cell reactivity in an ipilimumab-responsive melanoma. *Journal of Clinical Oncology*, *31*(32). https://doi.org/10.1200/JCO.2012.47.7521

Wang, T., Shao, W., Huang, Z., Tang, H., Zhang, J., Ding, Z., & Huang, K. (2021). MOGONET integrates multi-omics data using graph convolutional networks allowing patient classification and biomarker identification. *Nature Communications 12*, 3445. https://doi.org/10.1038/s41467-021-23774-w

Whalen, S., & Pandey, G. (2013). A comparative analysis of ensemble classifiers: Case studies in genomics. *Proceedings - IEEE International Conference on Data Mining, ICDM*, 807–816. https://doi.org/10.1109/ICDM.2013.21

Xu, K., Hu, W., Leskovec, J. & Jegelka, S. How powerful are graph neural networks? In Proceedings of the 7th International Conference on Learning Representations (ICLR 2019), 9104-9120 (OpenReview.net, 2019).

You, J., Ying, Z. & Leskovec, J. Design space for graph neural networks. In Advances in Neural Information Processing Systems 33 (eds Larochelle, H., Ranzato, M., Hadsell, R., Balcan, M.-F. & Lin, H.-T.) 17009-17021 (Curran Associates, Inc., 2020)
