# Supplementary material for: Immunotherapy drug target identification using machine learning and patient-derived tumour explant validation
Source: Nat Mach Intell. 2026 May 18;8(5):670–89. doi: 10.1038/s42256-026-01201-3 (PMC13201160; doi:10.1038/s42256-026-01201-3)
Supplement: Supplementary file 2 — Reporting Summary [file 42256_2026_1201_MOESM2_ESM.pdf]

nature portfolio

Corresponding author(s): Marcellus Augustine
Charles Swanton
Kevin Litchfield

Last updated by author(s): 17/12/2025

Reporting Summary

Nature Portfolio wishes to improve the reproducibility of the work that we publish. This form provides structure for consistency and transparency in reporting. For further information on Nature Portfolio policies, see our Editorial Policies and the Editorial Policy Checklist.

Statistics

For all statistical analyses, confirm that the following items are present in the figure legend, table legend, main text, or Methods section.

n/a	Confirmed	
☐	☒	The exact sample size (*n*) for each experimental group/condition, given as a discrete number and unit of measurement
☐	☒	A statement on whether measurements were taken from distinct samples or whether the same sample was measured repeatedly
☐	☒	The statistical test(s) used AND whether they are one- or two-sided<br>*Only common tests should be described solely by name; describe more complex techniques in the Methods section.*
☐	☒	A description of all covariates tested
☐	☒	A description of any assumptions or corrections, such as tests of normality and adjustment for multiple comparisons
☐	☒	A full description of the statistical parameters including central tendency (e.g. means) or other basic estimates (e.g. regression coefficient) AND variation (e.g. standard deviation) or associated estimates of uncertainty (e.g. confidence intervals)
☐	☒	For null hypothesis testing, the test statistic (e.g. *F*, *t*, *r*) with confidence intervals, effect sizes, degrees of freedom and *P* value noted<br>*Give P values as exact values whenever suitable.*
☒	☐	For Bayesian analysis, information on the choice of priors and Markov chain Monte Carlo settings
☒	☐	For hierarchical and complex designs, identification of the appropriate level for tests and full reporting of outcomes
☐	☒	Estimates of effect sizes (e.g. Cohen's *d*, Pearson's *r*), indicating how they were calculated

Our web collection on statistics for biologists contains articles on many of the points above.

Software and code

Policy information about availability of computer code

Data collection: For flow cytometry data, FACS Diva Software Version 9.1 (Symphony A5) was used.

Data analysis: All analysis performed using R v4.1.3 or Python v3.9

R packages
ggplot2 v3.4.1
ggpubr v0.6.0
tidyverse v2.0.0
tidyr v1.3.0
dplyr v1.1.0
reshape2 v1.4.1
readxl v1.4.3
data.table v1.15.4
matrixStats v1.3.0
Matrix.utils v0.9.8
magrittr v2.0.3
nortest v1.0.4
preprocessCore v1.56.0
limma v3.50.3
apeglm v1.16.0
DESeq2 v1.34.0

Seurat v4.3.0
SeuratObject v4.1.3
SingleCellExperiment v1.16.0
locfit v1.5-9.6
loomR v0.2.1.9
ACTIONet v2.1.9
SCINET v1.0
pROC v1.18.5
fmsb v0.7.6
WebGestaltR v0.4.6
ggraph v2.1.0
visNetwork v2.1.2
igraph v1.4.0

Python packages
pandas v1.4.1
numpy v1.23.5
joblib v1.2.0
iterative-stratification v0.1.6
sklearn v1.2.1
scikit-learn-intelex v2021.20211229.152138
imbalanced-learn v0.8.1
XGBoost v1.7.3
Optuna v3.0.3
Pytorch v2.0.0+cpu
Pytorch Geometric v2.3.1
shap v0.41.0
xswap v0.0.2

Software for demo GNNs (Mac and Linux):
python=3.9
pip
numpy=1.26
pandas=2.3
scikit-learn=1.4
imbalanced-learn=0.12
optuna=4.4
joblib
tqdm
pytz
python-dateutil
sqlalchemy
sqlite

Software for demo GNNs (Windows):
python=3.9
pip
numpy=1.26
pandas=2.3
scikit-learn=1.2.2
imbalanced-learn=0.12
optuna=3.0.3
joblib
tqdm
pytz
python-dateutil
sqlalchemy=1.4.44
sqlite

Build toolchain (needed to compile xswap)
compilers
cmake
make
git
llvm-openmp
wheel
setuptools

Software to analyse functional validation experimental data:
FACS Diva Software Version 9.1
FlowJo 10.10.0
BioLegend LegendPlex Qognit Cloud analysis software
GraphPad Prism Version 10.6.1

For manuscripts utilizing custom algorithms or software that are central to the research but not yet described in published literature, software must be made available to editors and reviewers. We strongly encourage code deposition in a community repository (e.g. GitHub). See the Nature Portfolio guidelines for submitting code & software for further information.

Data

Policy information about availability of data

All manuscripts must include a data availability statement. This statement should provide the following information, where applicable:

- Accession codes, unique identifiers, or web links for publicly available datasets
- A description of any restrictions on data availability
- For clinical datasets or third party data, please ensure that the statement adheres to our policy

Most datasets used herein are publicly available: single cell transcriptomics (see Methods for full details on individual studies), HLA-peptidomics (Bulik-Sullivan et al., 2018), CRISPR co-cultures (Vredevoogd et al., 2019; Lawson et al., 2020), GWAS catalog (Buniello et al., 2019), and Hetionet (Himmelstein and Baranzini, 2015). Bulk sequencing patient cohorts are described elsewhere: CPI1000+ (Litchfield et al., 2021), CPI3000+ (manuscript under preparation).

All relevant flow cytometry data are provided as Source or Supplementary Data.

Research involving human participants, their data, or biological material

Policy information about studies with human participants or human data. See also policy information about sex, gender (identity/presentation), and sexual orientation and race, ethnicity and racism.

Reporting on sex and gender	The sex and gender distribution of patients whose melanoma samples were used for patient-derived explants (PDEs) are as expected, given the small sample size (n=8) and the consecutive nature of recruitment.
Reporting on race, ethnicity, or other socially relevant groupings	All 8 melanoma patients were of European ancestry, consistent with the known epidemiology of melanoma.
Population characteristics	The median age was 68 (range 39-83), 63% were male. A variety of melanoma subtypes were included, such as cutaneous, unknown primary, acral and uveal subtypes. Tumours samples were derived from routine surgical resections of locoregional or distant metastatic sites. Most patients were naive to systemic therapy, 25% had prior anti-PD-1 in the adjuvant setting prior to tumour sample procurement.
Recruitment	Consecutive patients undergoing melanoma surgery at the Royal Marsden Hospital with excess tumour tissue remaining, following routine histopathological diagnostics, were recruited.
Ethics oversight	NHS Health Research Authority - London Chelsea Research Ethics Committee REC reference 11/LO/0003. IRAS:68421

Note that full information on the approval of the study protocol must also be provided in the manuscript.

Field-specific reporting

Please select the one below that is the best fit for your research. If you are not sure, read the appropriate sections before making your selection.

☒ Life sciences ☐ Behavioural & social sciences ☐ Ecological, evolutionary & environmental sciences

For a reference copy of the document with all sections, see nature.com/documents/nr-reporting-summary-flat.pdf

Life sciences study design

All studies must disclose on these points even when the disclosure is negative.

Sample size	No sample size calculations were performed. Sample sizes were selected based on available data and tissue.
Data exclusions	Pre-established exclusion criteria were applied in the following instances:<br>1. Removing all CD8+ T cells from models developed to predict the mutation-associated neoantigen score from single cell transcriptomics data<br>2. Excluding cell types identified as dying, normal, or unknown.
Replication	All the biological replicates in the experiments were performed independently. All attempts at replication were successful.
Randomization	Not applicable - not an interventional study
Blinding	Not applicable - not an interventional study

Reporting for specific materials, systems and methods

We require information from authors about some types of materials, experimental systems and methods used in many studies. Here, indicate whether each material, system or method listed is relevant to your study. If you are not sure if a list item applies to your research, read the appropriate section before selecting a response.

Materials & experimental systems

n/a	Involved in the study	
☐	☒	Antibodies
☒	☐	Eukaryotic cell lines
☒	☐	Palaeontology and archaeology
☒	☐	Animals and other organisms
☐	☒	Clinical data
☒	☐	Dual use research of concern
☒	☐	Plants

Methods

n/a	Involved in the study	
☒	☐	ChIP-seq
☐	☒	Flow cytometry
☒	☐	MRI-based neuroimaging

Antibodies

Antibodies used

Antibody used for flow cytometry (RRID, clone, dilution):
BUV395 Mouse Anti-Human Ki-67 (RRID: AB_2738577, Clone: B56, BD Biosciences, 1:40), BUV496 Mouse Anti-Human CD8 (RRID: AB_2870223, Clone RPA-T8, BD Biosciences, 1:80), BUV563 Mouse Anti-Human CD45RA (RRID: AB_2870211, Clone: HI100, BD Biosciences, 1:80), BUV737 Mouse Anti-Human CD39 (RRID: AB_2738919, Clone: TU66, BD Biosciences, 1:20), BUV805 Mouse Anti-Human CD3 (RRID: AB_2870181, Clone: SK7, BD Biosciences, 1:40), BV480 Mouse Anti-Human CD103 (RRID: AB_2743774, Clone: Ber-ACT8, BD Biosciences, 1:40), BV711 Mouse Anti-Human HLA-DR (RRID: AB_2738378, Clone: G46-6, BD Biosciences, 1:40), BB790-P Anti-Human CD4 (RRID: N/A, Clone: SK3, BD Custom Conjugates, 1:160), Alexa Fluor® 700 Mouse anti-Human Granzyme B (RRID AB_1645453, Clone: GB11, BD Biosciences, 1:80), Brilliant Violet 421™ Mouse anti-human CD279 (PD-1) (RRID: AB_10960742, Clone: SK3, BioLegend, 1:20), Brilliant Violet 650™ Mouse Anti-Human CD197 (CCR7) (RRID: AB_2563867, Clone: G043H7 , BioLegend, 1:10), Brilliant Violet 785™ Mouse Anti-Human CD45 (RRID: AB_2563129, Clone: HI30, BioLegend, 1:20), FITC Anti-Human HLA-A, -B, -C (RRID: AB_314873, Clone: W6/32, BioLegend, 1:40), PE Mouse Anti-Human TCF1 (TCF7) Antibody (RRID: AB_2728492, Clone: 7F11A10, BioLegend, 1:10), PE/Dazzle™ 594 Mouse Anti-Human CD137 (4-1BB) (RRID: AB_2566260, Clone: 4B4-1, BioLegend, 1:20), PE/Cyanine7 Mouse Anti-Human CD134 (OX40) (RRID: AB_10901161, Clone: Ber-ACT35, BioLegend, 1:20), APC Rat Anti-Human Foxp3 (RRID: AB_1603280, Clone: PCH101, Invitrogen, 1:40).

Validation

All antibodies were sourced commercially and used solely for applications validated by the manufacturer. Antibodies for flow cytometry were tested and optimised by staining both anti-CD3/-CD28 treated and untreated human Peripheral Blood Mononuclear Cells (PBMCs).

Clinical data

Policy information about clinical studies
All manuscripts should comply with the ICMJE guidelines for publication of clinical research and a completed CONSORT checklist must be included with all submissions.

Clinical trial registration

The study involved materials from TRAcking Cancer Evolution through therapy (Rx) (TRACERx) Melanoma: Exploratory analysis of genomic signatures of progression in melanoma (TRACERx Melanoma) (IRAS:68421). The study was reviewed and approved by both the Royal Marsden Committee for Clinical Research (CCR) (CCR:3569), and by London-Chelsea Research Ethics Committee (REC) (REC: 11/LO/0003), and was performed in compliance with all relevant ethical regulations.

Study protocol

This is not a clinical trial; there are no clinical interventions performed as part of this observational study. Study protocol can be provided upon request.

Data collection

Source clinical data are collected prospectively and stored securely within the Royal Marsden Hospital.

Outcomes

Exploratory objectives of TRACERx Melanoma are to identify novel molecular drivers of melanoma and immunotherapeutic targets.

Plants

Seed stocks

NA

Novel plant genotypes

NA

Authentication

NA

Flow Cytometry

Plots

Confirm that:

☒ The axis labels state the marker and fluorochrome used (e.g. CD4-FITC).

☒ The axis scales are clearly visible. Include numbers along axes only for bottom left plot of group (a 'group' is an analysis of identical markers).

☒ All plots are contour plots with outliers or pseudocolor plots.

☒ A numerical value for number of cells or percentage (with statistics) is provided.

Methodology

Sample preparation	Solid tumour lesions were macroscopically selected, by a pathologist, from the resected tumour material. The tumour was collected in ice-cold collection medium (University of Wisconsin Solution (Bridge to Life Ltd), supplemented with 100 µg/ml of Primocin (InvivoGen) for subsequent PDE cultures. Tissue materials collected for subsequent PDE cultures were immediately processed by manual cutting into tumour fragments (PDEs) of size 1-2 cubic millimetres on ice using a scalpel. After processing, PDEs from different tumour areas were mixed to ensure uniform representation of the tumour lesion and were cryopreserved in cryovials containing 1 ml FBS with 10% DMSO (Sigma) with 15 PDEs per vial. All PDEs were cryopreserved in liquid nitrogen (LN) until further usage.
Instrument	Standard flow cytometry was performed on BD FACSymohony A5 Cell Analyser (BD Biosciences).
Software	FACS Diva Software Version 9.1<br>FlowJo 10.10.0
Cell population abundance	No sorting was performed in this study.
Gating strategy	Sample clean-up was performed by gating on doublets exclusion in FSC-A/FSC-H, SSC-A/SSC-H and dead cell exclusion (eF780 LIVE/DEAD negative cells).<br>For T cell lineages, T cells were identified as CD45+/CD3+/CD8+ or CD45+/CD3+/CD4+/FoxP3-. Dysfunctional CD8+ T cells were identified as TCF-7- CD39+PD-1+ CD8+, pre-dysfunctional CD8+ T cells were identified as TCF-7+ CD39- PD-1+ CD8+.<br>For functional analysis, GZMB, PD-1, OX40, Ki-67, HLA-DR are analysed on both CD8+ T cells and FoxP3- CD4 T cells.

☒ Tick this box to confirm that a figure exemplifying the gating strategy is provided in the Supplementary Information.
